# Visible Light-Promoted Oxidative Cross-Coupling of Alcohols to Esters

**DOI:** 10.3390/molecules29030570

**Published:** 2024-01-23

**Authors:** Andrea Dellisanti, Elisa Chessa, Andrea Porcheddu, Massimo Carraro, Luisa Pisano, Lidia De Luca, Silvia Gaspa

**Affiliations:** 1 Dipartimento di Scienze Chimiche, Fisiche, Matematiche e Naturali, Università degli Sudi di Sassari, Via Vienna 2, 07100 Sassari, Italy; adellisanti@uniss.it (A.D.); elisachessa21@gmail.com (E.C.); mcarraro@uniss.it (M.C.); luisa@uniss.it (L.P.); sgaspa@uniss.it (S.G.); 2 Dipartimento di Scienze Chimiche e Geologiche, Università degli Sudi di Cagliari, 09042 Monserrato, Italy; porcheddu@unica.it

**Keywords:** ester, TCCA, visible light, photochemistry

## Abstract

Ester is one of the most significant functional groups in organic chemistry and is enclosed in several valued molecules. Usually, esters are prepared through the acid-catalyzed esterification reaction of carboxylic acids with alcohols, transesterification of esters with alcohols, or via activation of carboxylic acids followed by the addition of alcohols. However, these procedures typically imply the excess use of reactants and harsh reaction conditions. Visible light-mediated photoreactions have been disclosed to display a safe, sustainable, and accessible alternative to traditional methods, and to lead new reactivity modes in organic procedures. In this context, we propose a transition metal-based and organic-based photocatalyst-free synthesis of esters from alcohols induced by visible light. The methodology can be carried out using sunlight or artificial visible light as a solar simulator or a blue LED source.

## 1. Introduction

Esters are a very significant functional group in organic chemistry, and they are contained in several natural products, polymers, pharmaceuticals, and synthetic intermediates [1].

The most significant examples of molecules containing esters are biofuels such as biodiesel [2,3], solvents such as ethyl acetate and methyl acetate [4], pharmaceuticals [5], plastics and coatings [6,7], and herbicides and pesticides [8]. Moreover, esters are often used as flavoring components [9] and preservatives in food products [10], as additives in perfume [11], and as fragrances in the soap and cosmetic industry [12].

**Scheme 1:**
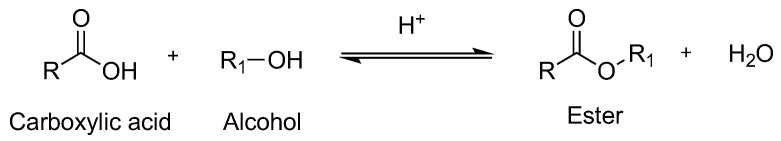
Fischer esterification.

Generally, esters are prepared via Fisher esterification, an acid-catalyzed esterification reaction between carboxylic acids and alcohols, which requires either of two methods to shift the equilibrium in favor of the products [13,14,15] (Scheme 1). One is the removal of water generated as the reactions proceed, via azeotropic distillation or using dehydrating agents, and the other is the use of large excess amounts of one of the reactants, usually alcohols. The reaction fails to achieve the completion, prejudicing the product yield; it is restricted by a slow rate of reaction and a low overall conversion because of the establishment of thermodynamic equilibrium. Moreover, conventional Fisher esterification has other complications associated with the generation of side reactions (such as oxidation and etherification), corrosion of the equipment, tedious purification procedures, and the discharge of large volumes of acidic wastes. Despite being industrially significant, the esterification process has yet to overcome these drawbacks in a cost-effective and environmentally friendly way [16].

**Scheme 2:**
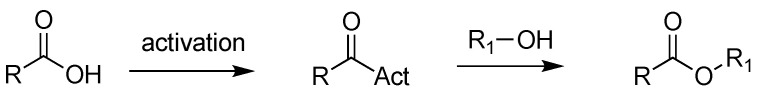
Synthesis of esters via activation of carboxylic acids.

Further, esters can be achieved through the succeeding activation of carboxylic acids (e.g., via acyl chlorides, anhydrides, and activated esters) [17], a process that converts the -OH of the acid into a good leaving group, followed by the addition of alcohols, under harsh reaction conditions [18] (Scheme 2). This procedure is commonly used for the industrial synthesis of pharmaceuticals containing esters, but it presents many limits, such as additional steps, the production of a stoichiometric amount of waste products, and the usage of hazardous reagents [19].

An appealing alternative approach is the oxidative esterification of aldehydes with alcohols, but aldehydes are usually gained by selective oxidation of alcohols [10,11,12,13,14,15,16,17,18,19,20,21,22].

**Scheme 3:**
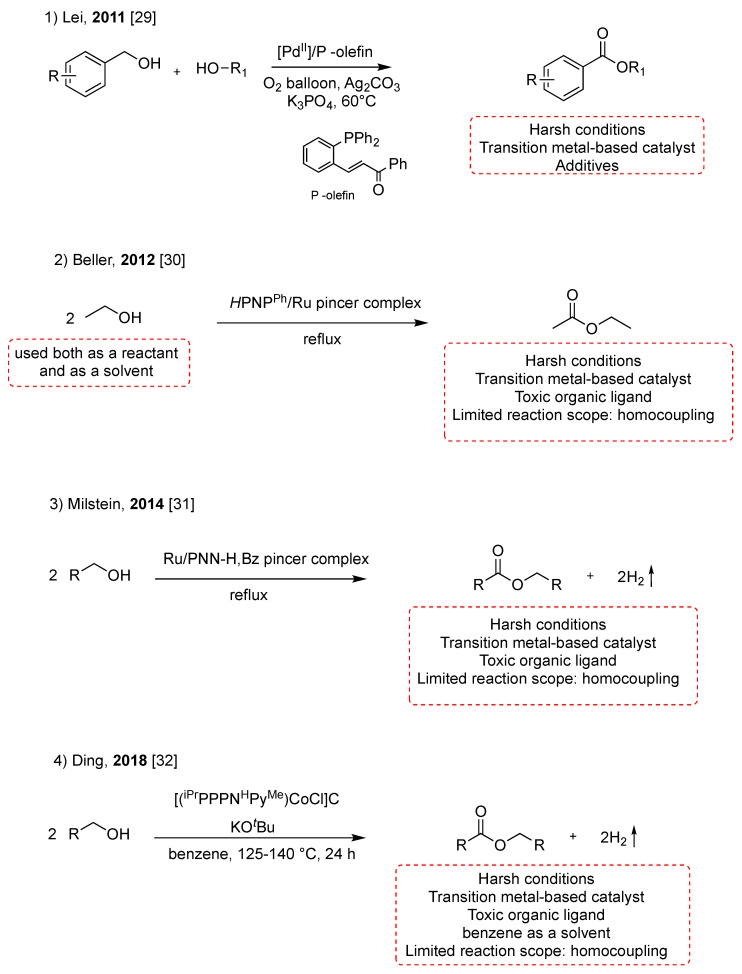
Synthesis of esters from alcohols [29,30,31,32].

Alcohols are easily accessible, stable, and abundant compounds and are present in many naturally occurring molecules. In light of these reasons, the direct conversion of alcohols to esters is a significant focus of green and sustainable chemistry and offers an economic approach to ester’s synthesis [23,24,25,26,27,28]. In recent years, the direct transformation of alcohols into esters has been achieved using catalysts based on metals such as Pd, Ru, and Co (Scheme 3). In 2011, Lei reported a palladium-catalyzed aerobic oxidative esterification of alcohols using a P-olefin ligand [29]. The reaction was achieved between benzylic alcohols with electron-donating substituents, such as Ph and OMe, and aliphatic alcohols with average yields of 50%, at 60 °C for 12 h (Scheme 3, pathway 1). The milestone of transition metal-catalyzed oxidative esterification of alcohols was Beller’s catalytic acceptorless dehydrogenation of ethanol to ethyl acetate using a Ru-PNP complex with TONs over 15,000 (Scheme 3, pathway 2) [30]. In 2014, Milstein reported a new class of phosphinopyridyl ruthenium pincer complexes, bearing sec-amine coordination, which catalyze homocoupling of alcohols to esters under mild conditions (Scheme 3, pathway 3).

The above methods require catalysts prepared with expensive precious toxic transition metals and toxic ligands, with consequent increases in the cost and challenges for the subsequent separation and purification of products. The ruthenium complexes were obtained by a two-step synthesis carried out at, respectively, 100 °C for 12 h and 65 °C for 4 h [31].

Later, Ding’s group developed a catalytic system with a tripodal cobalt complex supported by a tetradentate tripodal ligand to achieve the homocoupling of primary alcohols to esters in benzene as a solvent for 24 h at 120 °C (Scheme 3, pathway 4) [32]. Although catalysts based on non-noble metals have been proposed and studied, these catalytic systems still need many organic ligands or high pressure.

Recently, single-side organically decorated Anderson-type chrome-based catalyst [N(C_4_H_9_)_4_]_3_[CrMo_6_O_18_(OH)_3_{(OCH_2_)_3_CCH_2_OH}] to efficiently convert alcohols to esters has been reported [33]. The catalyst has been prepared by reacting (NH_4_)_6_Mo_7_O_24_·2H_2_O with Cr(NO_3_)_3_ to obtain (NH_4_)_3_[Cr(OH)_6_Mo_6_O_18_]. The compound (NH_4_)_3_[Cr(OH)_6_Mo_6_O_18_] was treated with pentaerythritol under reflux at 100 °C for 12 h, and the desired Anderson-type chrome-based catalyst was obtained by adding tetrabutyl bromide to the solution in batches. This pentaerythritol-decorated Anderson-type polyoxometalate has been employed to catalyze the conversion of alcohols into esters in the presence of 3 mmol H_2_O_2_ used as an oxidant at 65 °C for 36 h.

**Scheme 4:**
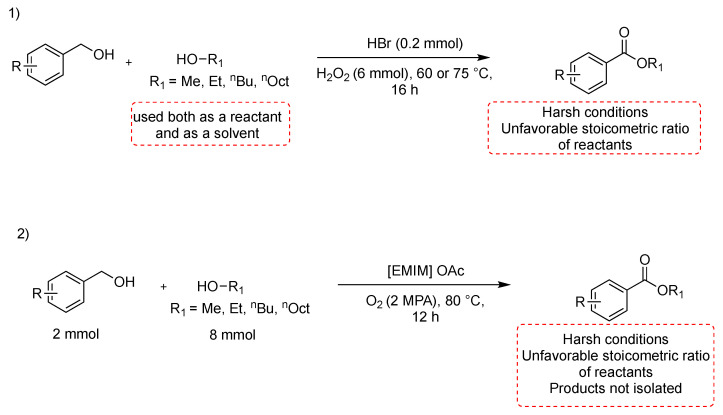
Metal-free esterification of alcohols.

Metal-free oxidative esterification of alcohols is infrequently reported in the literature, even if it is of particular importance to the separation of a metal catalyst from products. Furthermore, transition metal-catalyzed reactions also produce hazardous waste, which is environmentally problematic and, hence, should be avoided wherever possible. Furthermore, it is also highly desirable to develop environmentally benign oxidative esterification processes without the use of any metal-based catalyst. A transition metal-free oxidative esterification of benzylic alcohols with catalytic amount of HBr in aqueous medium at 60–75 °C for 16 h has been reported [34]. The reaction proceeds in the presence of a large excess of the oxidant, and the alcohol is used as a solvent. The methodology is suitable for substituted benzylic alcohols, which have been esterified with methanol, ethanol, *n*-butanol, and *n*-octanol; it is also suitable for selective mono-esterification of ethylene glycol and glycerol (Scheme 4, path 1). Another interesting route to esters from alcohols consists of a cross-esterification between benzyl alcohols and aliphatic alcohols in the basic ionic liquid 1-ethyl-3-methylimidazolium acetate ([EMIM] OAc), used both as a solvent and as a catalyst (Scheme 4, path 2) [35]. The benzyl alcohol (2 mmol) is reacted with aliphatic alcohol (8 mmol) in [EMIM]OAc, at 80 °C for 12 h. IL_S_ need to be dried under vacuum at 80 °C for 24 h to remove water and organic impurities, which otherwise might interfere with the catalytic activity. The IL_S_ can be reused five times by being dried under a vacuum at 50 °C for 24 h after the extraction process. Unfortunately, when two different benzylic alcohols were employed as substrates, a mixture including their self- and cross-esterification products was generated.

These methodologies are oriented to the preparation of methyl benzoate and its analogs. Methyl benzoate is a volatile organic compound that is present naturally as a floral aroma in many plants and is capable of killing insects at different life stages. Compared with commercial pesticides, methyl benzoate is 1.3 to 3.4 times more toxic to gypsy moth larvae and brown marmorated stinkbug nymphs. It is believed to be more desirable as an insect pest toxin than a conventional synthetic insecticide due to its rapid environmental biodegradable property and potentially lower toxicity against natural enemies, humans, and other mammals. Methyl benzoate and benzoate esters are an appealing green alternative to commercially available arthropod repellents based on *N*, *N*-diethyl-3-methylbenzamide (DEET) [36]. Moreover, methyl benzoate and benzoate esters are widely used in the perfume industry [37]. They are also an important class of chemical intermediates, commonly used in plasticizers, pharmaceuticals, dyeing polyester fibers, solvents, and disinfectants [38,39,40].

Although the synthesis of benzoate esters is an important target, unfortunately, these procedures suffer from unfavorable stoichiometric ratios of the reagent and hard reaction conditions. Moreover, the use of transition metal-based catalysts, which need to be synthesized, limits the accessibility of these methodologies and their improvement in view of industrial-scale applications. It is, thus, desirable to explore the prospect of finding a new, more sustainable, easily and directly accessible, and cheaper route. Therefore, the development of a new methodology, driven by a green energy source, for effective conversion of alcohols to esters would be highly desirable. One of the most relevant research topics in organic chemistry is the development of new methodologies induced by visible light [41,42]. Photosynthesis can be defined as the transformation of light into chemical energy, efficiently used to promote chemical synthesis, allowing for the development of sustainable and efficient synthetic methodologies. Visible light can be considered a clean reagent: it activates the substrates leaving no residues in the subsequent mixture, with considerable exemplification of the work-up and purification operations. Moreover, the use of visible light, instead of thermal energy, for performing chemical processes, permits great energy savings. For these reasons, visible light-mediated organic synthesis has reached strong urgency because of the development of sustainable chemistry methods [43]. In addition, the majority of photochemical reactions require catalysts based on heavy or rare metals, such as ruthenium and iridium, or organic photocatalysts. So, a photoreaction, which arises without any catalyst, represents an interesting aim. Due to our interest in the oxidative transformations of alcohols [44], we studied the possibility of directly converting primary benzylic alcohols into esters using trichloroisocyanuric acid (TCCA) under visible light irradiation. TCCA is an inexpensive, commercially available, nontoxic, versatile, and efficient oxidizing reagent and chlorinating [45] reagent, frequently used for the disinfection of swimming pools, dishware, houses, hotels, and public places, as well as in fruit and vegetable preservation [46]. The selected reagents have several advantages: alcohols are stable, readily accessible, inexpensive, and commercially available compounds and trichloroisocyanuric acid is a safe, easy-to-handle, and shelf-stable solid. Moreover, the use of visible light allows for a display of a safe, sustainable, and accessible alternative to traditional methods, and the introduction of new reactivity modes in organic synthesis.

## 2. Results and Discussion

### 2.1. Study of the Methodology and Optimization Conditions

**Scheme 5:**
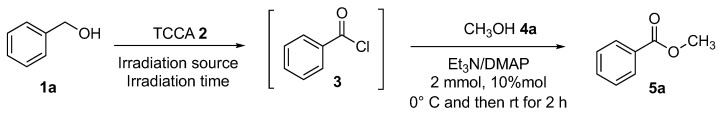
General scheme of the transformation of alcohols to esters.

We started our investigation by reacting benzyl alcohol **1a** (1.1 mmol) and TCCA **2** (1.5 mmol) in dichloromethane (2 mL) under solar simulator irradiation (Scheme 5) for 1 h (until the complete transformation of the starting alcohol **1a**). The resultant benzoyl chloride **3** was quantitatively formed. The resulting mixture, containing the acyl chloride generated in situ, was added with NEt_3_ (2 mmol) in the presence of a catalytic amount of DMAP (10 mol%), and after 2 h at room temperature, the desired ester was formed in 67% yield (Table 1, **5a**, entry 1). Several process parameters have been investigated and the whole optimization process is summarized in Table 1. For the purpose of optimizing the product yield, the reduction of the amount of TCCA **2** was investigated. The same reaction was carried out employing 1.2 mmol (Table 1, entry 2) and 1.3 mmol (Table 1, entry 3) of TCCA **2**, and the ester **5a** was achieved in 77% and 80%, respectively. Usually, photochemical-mediated methodologies are not clean and they are carried out at a low concentration (>1 M) in order to reduce the secondary photoreactions and, in general, to minimize the product distribution [47]. So, the amount of the solvent was doubled (Table 1, entry 4) and the product yield reached 87%. Then the irradiation time was increased to 1.5 h (Table 1, entry 5), affording an increase in yield of up to 99%. In order to reduce the overall energy expenditure requirement, natural sunlight was employed as an alternative green energy source. The use of solar energy is a favorable tool because it is a reliable, renewable, and advantageous source of energy [42]. Then the synthesis of esters was performed under sunlight irradiation, giving comparable results as reported in Table 1, entry 6. In order to extend the applicability, reliability, and reproducibility of the methodology, the use of blue LED was explored, and methyl benzoate **5a** was found in 99% yield (Table 1, entry 7). A screening of solvents was carried out: cyclopentyl methyl ether (CPME) (Table 1, entry 9), tetrahydrofuran (THF) (Table 1, entry 10), acetonitrile (Table 1, entry 11), and ethyl acetate (Table 1, entry 12) were tested, but no formation of methyl benzoate **5a** was detected. Using dichloroethane (Table 1, entry 13) as a solvent, ester **5a** was obtained, but only in trace amounts. The same transformation was carried out in the dark and in the absence of TCCA, but no product formation was observed (Table 1, entry 14).

### 2.2. Investigation of Reaction Applicability

**Scheme 6:**
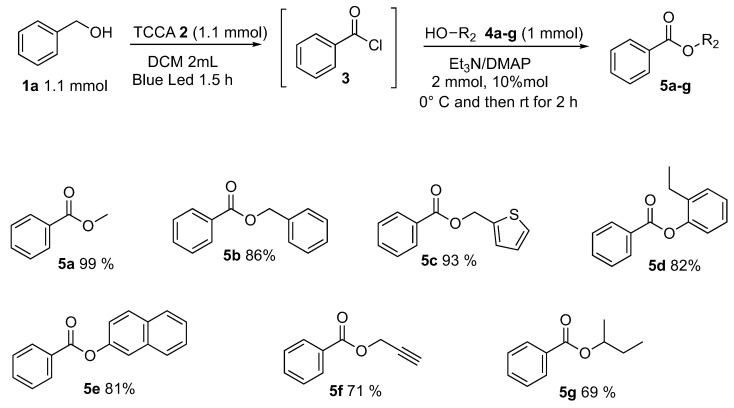
Alcohol’s scope.

After the optimized conditions were fixed (Table 1, entry 8), the applicability and functional group tolerance were explored. An array of alcohols R_2_OH, keeping the reactant **1a** fixed (Scheme 6), were employed and tested. Benzyl alcohol and thiophen-2-methanol furnished the corresponding esters **5b** and **5c** in very good yields—respectively, 86% and 93%. In order to test the applicability of the methodology, less nucleophilic phenolic alcohols, 2-ethylphenol, and naphthalen-1-ol were reacted. We were delighted to observe that phenols, which are weak nucleophiles, reacted successfully delivering the 2-ethylphenyl benzoate **5d** and 2-ethylphenyl benzoate **5e** in a satisfying way (82% and 81% yields). Even if the TCCA is a chlorinating reagent, the esterification showed excellent compatibility toward triple bond and prop-2-yn-1-ol furnished the corresponding prop-2-yn-1-yl benzoate **5f** in 71% yield. A sterically hindered secondary alcohol, butan-2-ol, was reacted following optimized conditions affording sec-butyl benzoate **5g** in 69% yield.

**Scheme 7:**
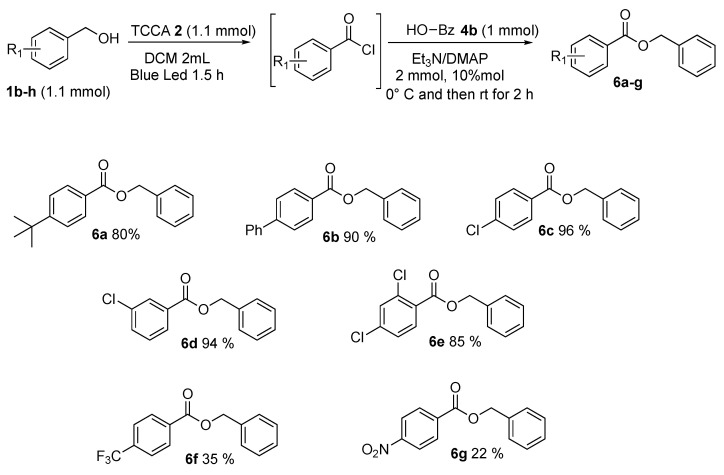
Benzyl alcohol’s scope.

Then, the benzyl alcohols **1b**–**h** were investigated (Scheme 7). In general, the corresponding esters were gained in good yields (Scheme 7, **6a**–**g**). Various functional groups on aromatic rings, both electron-donating and electron-withdrawing, were studied. The steric effects of substituents on the ring of substituted benzyl alcohols were found to have no effect on the yields. Instead, the electronic properties of substituents affected the results. Electron-donating groups in para position as *t*-butyl and phenyl showed very good results and benzyl 4-(tert-butyl)benzoate **6a** and benzyl [1,1′-biphenyl]-4-carboxylate **6b** were found in, respectively, 80% and 90% yields. Benzyl alcohol with halide substituents, such as chlorine in para, meta, and orto, para positions was subjected to this procedure producing the resultant esters **6c**, **6d,** and **6e** in very satisfactory yields, which could be further transformed by traditional cross-couplings. Strong electron-withdrawing groups, such as CF_3_ and NO_2_, provided benzyl 4-(trifluoromethyl)benzoate **6f** and benzyl 4-nitrobenzoate **6g**, in poor yields—respectively, 35% and 22%.

**Scheme 8:**
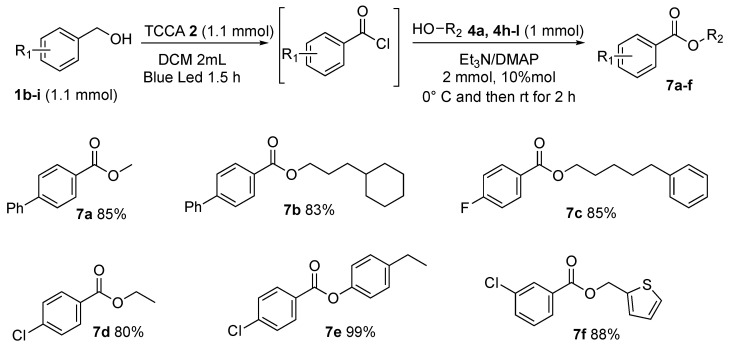
General scope.

Finally, for the purpose of exploring the general applicability of the procedure, variously substituted benzyl alcohols were reacted with an array of aliphatic alcohols, benzylic alcohols, and phenols (Scheme 8, **7a**–**f**). Of particular interest are the esters composed of alkyloxy chains of medium length, such as 3-cyclohexylpropyl [1,1′-biphenyl]-4-carboxylate **7b**, and 5-phenylpentyl 4-fluorobenzoate **7c**, found in, respectively, **83%** and **85%** yields. To prove the synthetic utility of the procedure, thiophen-2-ylmethanol was subjected to optimized conditions, producing the desired heteroaryl ester thiophen-2-ylmethyl 3-chlorobenzoate **7f** in 88% yield.

### 2.3. Proposed Mechanism

**Scheme 9:**
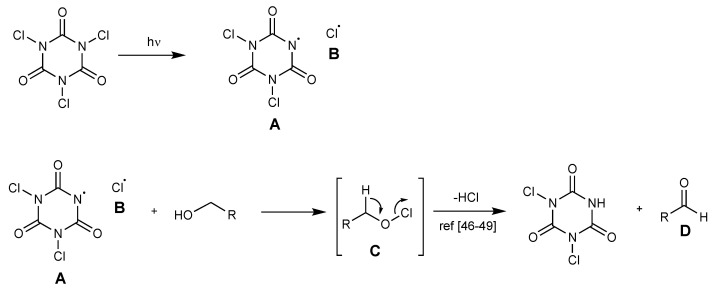
Proposed mechanism for aldehyde formation.

A plausible reaction mechanism has been fixed. The process is supposed to be a radical mechanism. It initiates from a visible light-assisted homolytic cleavage of the N–Cl bond in TCCA to form amidyl radical [48,49,50] **A** and radical chlorine atom **B**. Alcohol is converted into a hypochlorite compound **C**, which readily loses hydrogen chloride to form aldehyde **D** [51,52,53] (Scheme 9).

**Scheme 10:**
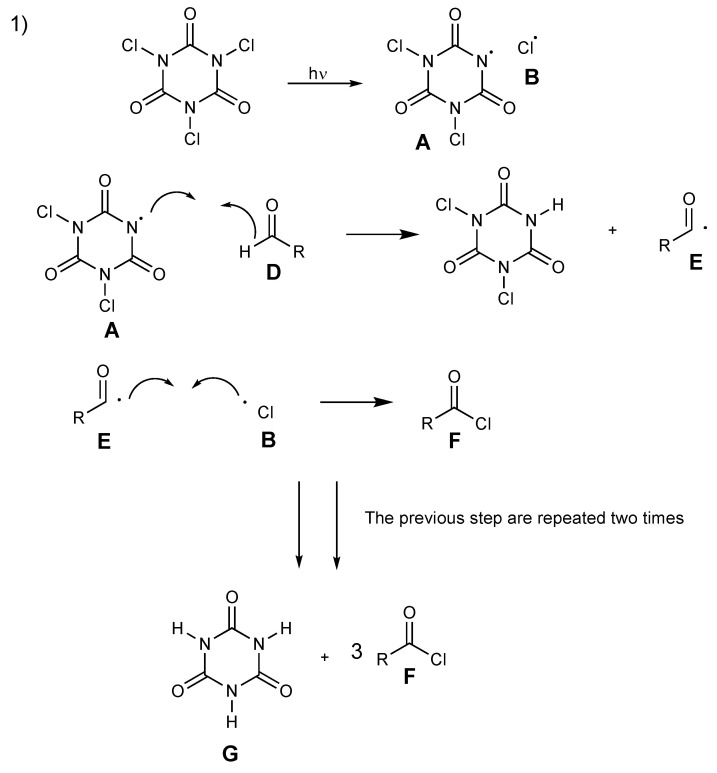
Proposed mechanism for acyl chloride formation, pathway 1.

**Scheme 11:**
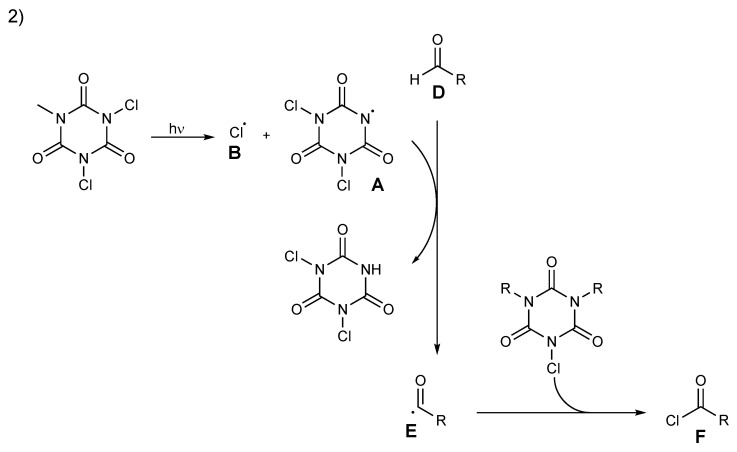
Proposed mechanism for acyl chloride formation, pathway 2.

Two plausible pathways of the mechanism for the formation of acyl chlorides from aldehydes with TCCA are shown in Scheme 10 and Scheme 11. In the first possible pathway (Scheme 10), N-centered radical **A** abstracts a hydrogen atom at the aldehyde **D** to form acyl radical **E**. The intermediary acyl radical **E** and chlorine radical **B** react together to form acyl chloride **F**. These key steps are repeated until the generation of cyanuric acid **G** allows for the complete reaction of all chlorine atoms.

An alternative plausible pathway, pathway 2, is proposed in Scheme 11. The reaction starts with a homolytic cleavage of the N–Cl bond of TCCA to generate radical chlorine atom **B** and nitrogen-centered radical **A** and then propagates via abstraction of aldehydes’ hydrogen by **A** to generate intermediate acyl radical **E**. Quenching of the acyl radical **E** by chloride transfer produces acyl chloride **F** and, subsequently, proceeds until the halogen source has been consumed (Scheme 11).

**Scheme 12:**
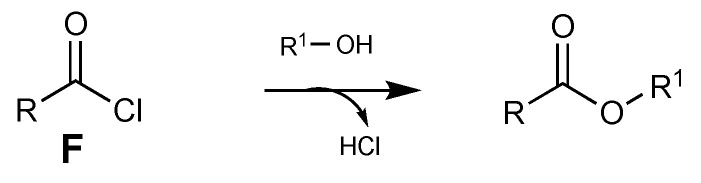
Addition of alcohol to acyl chloride.

Finally, the acyl chloride **F** reacts with alcohol to yield the desired ester (Scheme 12) [54].

## 3. Materials and Methods

### 3.1. Materials

All solvents and reagents were employed as acquired by commercial providers. All the reactions were carried out in an Argon atmosphere using standard procedures. All solvents were dried by common methods and distilled in an Argon atmosphere. Column chromatography was generally executed on silica gel (pore size 60 Å, 32–63 nm particle size) and reactions were monitored by thin-layer chromatography (TLC); analysis was accomplished with Merck Kieselgel 60 F254 plates and visualized using UV light at 254 nm, KMnO_4_, 2,4-DNP, and cerium ammonium molybdate staining. The reactions were carried out with the Abet tech sun 2000 simulator (under 100 mW/cm^2^ simulated AM 1.5G irradiance). Irradiation with blue light was performed with OSRAM Oslon SSL 80 LDCQ7P-1U3U (blue, λ max = 455 nm, I max = 1000 mA, 1.12 W). ^1^H NMR and ^13^C NMR spectra were recorded by a Bruker Avance III 400 spectrometer (400 MHz or 100 MHz, respectively) using CDCl_3_ solutions and TMS as an internal standard. Chemical shifts are indicated as parts per million (ppm, d) relative to the internal tetramethylsilane standard (TMS, d 0.00). The peak patterns are reported as follows: s, singlet; d, doublet; t, triplet; m, multiplet; q, quartet; dd, doublet of doublets; br, broad. The coupling constants, J, are indicated in Hertz (Hz). High-resolution mass spectra HRMS (HESI-FT-ORBITRAP) were performed on a Q-Exactive Thermo Scientific mass spectrometer (Waltham, MA, USA). Melting points were recorded in open capillary tubes and were uncorrected.

### 3.2. General Procedure for the Preparation of Methyl Benzoate ***5a*** via Solar Simulator Irradiation

In a round bottom flask of 10 mL, furnished by a condenser, TCCA (1.3 mmol) was added to benzyl alcohol (1.1 mmol) solved in 2 mL dichloromethane, in an Ar atmosphere and at room temperature. The resultant suspension was stirred and irradiated by the solar simulator for 1.5 h in an Ar atmosphere (the progress of the synthesis was monitored by TLC until the disappearance of starting benzyl alcohol, **1a**). Afterward, the obtained mixture was cooled to 0 °C and stirred under an inert atmosphere of dry argon. First, methanol (1.0 mmol) was added dropwise via syringe, then NEt_3_ (2.0 mmol) was added dropwise, and finally DMAP (10% mol) at once. After completion of the addition, the resulting mixture was stirred at room temperature until the disappearance of the methanol (monitored by TLC, the reaction is usually complete in about 1 h). Afterward, the solvent was removed in a vacuum and the residue was purified by flash chromatography. Pale yellow oil was obtained (0.135 g, 99% yield).

### 3.3. General Procedure for the Preparation of Methyl Benzoate ***5a*** via Sunlight Irradiation

In a round bottom flask of 10 mL, furnished by a condenser, TCCA (1.3 mmol) was added to benzyl alcohol (1.1 mmol) solved in 2 mL dichloromethane, in an Ar atmosphere and at room temperature. The resultant suspension was stirred and irradiated by sunlight for 1.5 h, under an Ar atmosphere (the progress was monitored by TLC until the disappearance of the starting benzyl alcohol, **1a**). Afterward, the resulting mixture was cooled to 0 °C and stirred under an inert atmosphere of dry argon. First, methanol (1.0 mmol) was added dropwise via syringe, then NEt_3_ (2.0 mmol) was added dropwise, and finally DMAP (10% mol) at once. After completion of the addition, the obtained mixture was stirred at room temperature until the disappearance of the methanol (monitored by TLC, the reaction is usually complete in about 1 h). Then the solvent was removed under reduced pressure, and the residue was purified by flash chromatography. Pale yellow oil was obtained (0.134 g, 98% yield).

### 3.4. General Procedure for the Synthesis of Esters ***5a**–**g***, ***6a**–**g*** and ***7a**–**f*** via Blue LED Irradiation

In a round bottom flask of 10 mL, furnished by a condenser, TCCA (1.1 mmol) was added to benzyl alcohol **1a** (1.1 mmol) solved in 1 mL dichloromethane, under an Ar atmosphere, at room temperature. The resultant suspension was stirred and irradiated with blue LED (455 nm) for 1.5 h at 25 °C, in an Ar atmosphere (the progress of the reaction was monitored by TLC until the disappearance of benzyl alcohol, **1a**). Afterward, the resultant mixture was cooled to 0 °C and stirred under an inert atmosphere of dry argon. First, alcohol (1.0 mmol) was added dropwise via syringe, then NEt_3_ (2.0 mmol) was added dropwise, and finally DMAP (10% mol) at once. After completion of the additions, the resulting mixture was stirred at room temperature until the disappearance of the starting alcohol (monitored by TLC, the reaction is generally complete in about 1 h). Then, the solvent was removed under the reduced pressure, and the residue was purified by flash chromatography.

### 3.5. Detection and Isolation of Benzoyl Chloride ***3***

In a round bottom flask of 10 mL, furnished by a condenser, TCCA (2.2 mmol) was put in a solution of benzyl alcohol, **1a** (2.2 mmol), and 2 mL dichloromethane under an Ar atmosphere, at room temperature. The resultant suspension was stirred under blue LED irradiation (455 nm) for 1.5 h in an Ar atmosphere (the progress of the synthesis was monitored by TLC until the disappearance of the starting reactant). The mixture of the reaction was filtered on Celite, then the solvent was removed under the reduced pressure, and the residue was distilled.

## 4. Conclusions

In conclusion, a visible light-promoted cross-coupling of alcohols to esters was reported. The starting materials have several advantages: alcohols are stable, readily accessible, inexpensive, and commercially available compounds; trichloroisocyanuric acid is a safe, easy-to-handle, shelf-stable solid; and the use of visible light as a source of energy is very appealing from an ecological point of view. The conditions are mild, and the stoichiometric ratio of the reagents is ideal. The methodology has shown good versatility and applicability, and various functional groups are well tolerated, providing an alternative approach to visible light-mediated ester synthesis, which avoids the use of expensive and toxic transition metal-based catalysts and toxic organic ligands. The reaction can be carried out using different visible light sources, such as blue LED, solar simulator, and sunlight. The methodology is operationally simple and the reactants as well as the visible light sources are economical and readily accessible. The proposed procedure appears to be very suitable to prepare benzoate esters, which are an industrially relevant class of compounds.

## Figures and Tables

**Table 1:** Screening of conditions for the esterification of **1a** to **5a**.

Entry	Irradiation Source	Irradiation Time	TCCA (mmol)	Solvent	Yield ^1^ [%]
1	Solar simulator	1	1.1	DCM (1 mL)	67%
2	Solar simulator	1	1.2	DCM (1 mL)	77%
3	Solar simulator	1	1.3	DCM (1 mL)	80%
4	Solar simulator	1	1.3	DCM (2 mL)	87%
5	Solar simulator	1.5	1.3	DCM (2 mL)	99%
6	Sun	1.5	1.3	DCM (2 mL)	98%
7	Blue LED	1.5	1.3	DCM (2 mL)	99%
8	Blue LED	1.5	1.1	DCM (2 mL)	99%
9	Blue LED	1.5	1.1	CPME (2 mL)	-
10	Blue LEDd	1.5	1.1	THF (2 mL)	-
11	Blue LED	1.5	1.1	CH_3_CN (2 mL)	-
12	Blue LED	1.5	1.1	AcOEt (2 mL)	-
13	Blue LED	1.5	1.1	DCE (2 mL)	Trace
14 ^2^	None	1.5	1.1	DCM (2 mL)	-

All the reactions were carried out with the same experimental parameters unless otherwise specified: Starting alcohol **1a** (1.1 mmol) in the solvent was reacted with TCCA **2** under light irradiation. Then, the resulting mixture was cooled to 0 °C, and MeOH (1.0 mmol) NEt_3_ (2.0 mmol) and then DMAP (10% mol) were added, and after 2 h at room temperature, the desired ester **5a** was obtained. ^1^ Isolated yields. ^2^ Reaction in the dark.

## Data Availability

The data presented in this study are available in the Supplementary Materials.

## Supplementary Materials

The following supporting information can be downloaded at: https://www.mdpi.com/article/10.3390/molecules29030570/s1, General information; General procedure for the synthesis of methyl benzoate **5a** under solar simulator irradiation; General procedure for the synthesis of methyl benzoate **5a** under sunlight irradiation; General procedure to esters under blue LED irradiation; Compound characterizations; Detection and characterization of benzoyl chloride 3; References [25,55,56,57,58,59,60,61,62,63,64,65,66,67,68,69,70,71,72,73]; NMR spectra of esters; NMR spectra of benzoyl chloride (3); UV-Vis spectra.

## References

[1] Ishihara K. (2009). Dehydrative condensation catalyses. Tetrahedron.

[2] Moser B.R. (2011). Biodiesel Production, Properties, and Feedstocks. Biofuels.

[3] Ambat I., Srivastava V., Sillanpaa M. (2018). Recent advancement in biodiesel production methodologies using various feedstock: A review. Renew. Sustain. Energy Rev..

[4] Petibon R., Aiken C.P., Ma L., Xiong D., Dahn J.R. (2015). The use of ethyl acetate as a sole solvent in highly concentrated electrolyte for Li-ion batteries. Electrochim. Acta.

[5] De Nazare de Oliveira A., de Oliveira D.T., Angèlica R.S., de Aguiar Andrade E.H., do Rosàrio da Silva J.K., da Rocha Filo G.N., Coral N., de Oliveira Pires L.H., Luque R., Santos do Nascimento L.A. (2020). Efficient esterification of eugenol using a microwave-activated waste kaolin. React. Kinet. Mech. Catal..

[6] Pereira C.S., Silva V.M., Rodrigues A.E. (2011). Ethyl lactate as a solvent: Properties, applications and production processes—A review. Green Chem..

[7] Tang X., Chen E.Y.-X. (2019). Toward Infinitely Recyclable Plastics Derived from Renewable Cyclic Esters. Chem.

[8] Zhang W., Jiang W. (2020). Antioxidant and antibacterial chitosan film with tea polyphenols-mediated green synthesis silver nanoparticle via a novel one-pot method. Int. J. Biol. Macromol..

[9] Zare M., Golmakani M.-T., Sardarian A. (2020). Green synthesis of banana flavor using different catalysts: A comparative study of different methods. Green Chem. Lett. Rev..

[10] De Barros D.P.C., Azevedo A.M., Cabral J.M.S., Fonseca L.P. (2012). Optimization of flavor esters synthesis by *Fusarium solani pisi* cutinase. J. Food Biochem..

[11] Almeida S.A.A.G., de Meneses A.C., de Araùjo P.H.H., de Oliveira D. (2017). A review on enzymatic synthesis of aromatic esters used as flavor ingredients for food, cosmetics and pharmaceuticals industries. Trends Food Sci. Technol..

[12] Steele J.H., Bozor M.X., Boyce G.R. (2020). Transumtation of scent: An evaluation of the synthesis of methyl cinnamate, a commercial fragrance, via a Fischer esterification for the second-year organic laboratory. J. Chem. Educ..

[13] Otera J., Dan-oh N., Nozaki H. (1991). Novel template effects of distannoxane catalysts in highly efficient transesterification and esterification. J. Org. Chem..

[14] Ishihara K., Ohara S., Yamamoto H. (1996). 3,4,5-Trifluorobenzeneboronic Acid as an Extremely Active Amidation Catalyst. J. Org. Chem..

[15] Storck S., Maier W.F., Salvado I.M.M., Ferreira J.M.F., Guhl D., Souverijins W., Martens J.A. (1997). Amorphous Sn/Si Mixed Oxides, Mild Solid Lewis Acid Catalysts for Esterification and Etherification Reactions. J. Catal..

[16] Mustafa A., Faisal S., Ahmed I.A., Munir M., Pereira Cipolatti E., Manoel E.A., Pastore C., di Bitonto L., Hanelt D., Nitbani F.O. (2023). Has the time finally come for green oleochemicals and biodiesel production using large-scale enzyme technologies? Current status and new developments. Biotechnol. Adv..

[17] Otera J., Nishikido J. (2010). Esterification: Methods, Reactions, and Applications.

[18] Wipf P. (2005). Handbook of Reagents for Organic Synthesis.

[19] Valeur E., Bradley M. (2009). Amide bond formation: Beyond the myth of coupling reagents. Chem. Soc. Rev..

[20] Enache D.I., Edwards J.K., Landon P., Solsona-Espriu B., Carley A.F., Herzing A.A., Watanabe M., Kiely C.J., Knight D.W., Hutchings G.J. (2006). Solvent-free oxidation of primary alcohols to aldehydes using Au-Pd/TiO_2_ catalysts. Science.

[21] Mallat T., Baiker A. (2004). Oxidation of alcohols with molecular oxygen on solid catalysts. Chem. Rev..

[22] Liu C., Tang S., Zheng L., Liu D., Zhang H., Lei A. (2012). Covalently Bound Benzyl Ligand Promotes Selective Palladium-Catalyzed Oxidative Esterification of Aldehydes with Alcohols. Angew. Chem. Int. Ed..

[23] Tang S., Yuan J., Liu C., Lei A. (2014). Direct oxidative esterification of alcohols. Dalton Trans..

[24] Ekoue-Kovi K., Wolf C. (2008). One-pot oxidative esterification and amidation of aldehydes. Chem. Eur. J..

[25] Feng J., Liang S., Chen S.-Y., Zhang J., Fu S.-S., Yu X.-Q. (2012). A Metal-Free Oxidative Esterification of the Benzyl C–H Bond. Adv. Synth. Catal..

[26] Liu C., Tang S., Lei A. (2013). Oxidant controlled Pd-catalysed selective oxidation of primary alcohols. Chem. Commun..

[27] Wang J., Liu C., Yuan J., Lei A. (2013). Transition-metal-free aerobic oxidation of primary alcohols to carboxylic acids. New J. Chem..

[28] Xia J., Shao A., Tang S., Gao X., Gao M., Lei A. (2015). Palladium-catalysed oxidative cross-esterification between two alcohols. Org. Biomol. Chem..

[29] Liu C., Wang J., Meng L., Deng Y., Li Y., Lei A. (2011). Palladium-Catalyzed Aerobic Oxidative Direct Esterification of Alcohols. Angew. Chem. Int. Ed..

[30] Nielsen M., Junge H., Kammer A., Beller M. (2012). Towards a Green Process for Bulk-Scale Synthesis of Ethyl Acetate: Efficient Acceptorless Dehydrogenation of Ethanol. Angew. Chem. Int. Ed..

[31] Fogler E., Garg J.A., Hu P., Leitus G., Shimon L.J.W., Milstein D. (2014). System with Potential Dual Modes of Metal–Ligand Cooperation: Highly Catalytically Active Pyridine-Based PNNH–Ru Pincer Complexes. Chem. Eur. J..

[32] Paudel K., Pandey B., Xu S., Taylor D.K., Tyer D.L., Lopez Torres C., Gallagher S., Kong L., Ding K. (2018). Cobalt-Catalyzed Acceptorless Dehydrogenative Coupling of Primary Alcohols to Esters. Org. Lett..

[33] Wang J., Jiang F., Tao C., Yu H., Ruhlmann L., Wei Y. (2021). Oxidative esterification of alcohols by a single-side organically decorated Anderson-type chrome-based catalyst. Green Chem..

[34] Samanta S., Pappula V., Dinda M., Adimurthy A. (2014). Transition metal-free oxidative esterification of benzylic alcohols in aqueous medium. Org. Biomol. Chem..

[35] Liu M., Zhang Z., Liu H., Xie Z., Mei Q., Han B. (2018). Transformation of alcohols to esters promoted by hydrogen bonds using oxygen as the oxidant under metal-free conditions. Sci. Adv..

[36] Feng Y., Chen J., Zhang A. (2018). Commercially available natural benzyl esters and their synthetic analogs exhibit different toxicities against insect pests. Sci. Rep..

[37] Aggarwal P., Dollimore D., Alexander K. (1997). The use of thermogravimetry to follow the rate of evaporation of an ingredient used in perfumes. J. Ther. Anal. Calorim..

[38] Wardzińska E., Penczek P. (2005). Influence of the glycol component in dibenzoate plasticizers on the properties of plasticized PVC films. J. Appl. Polym. Sci..

[39] Tekin E., de Gans B.J., Schubert U.S. (2004). Ink-jet printing of polymers–from single dots to thin film libraries. J. Mater. Chem..

[40] Patel J.P., Deshmukh S., Zhao C., Wamuo O., Hsu S.L., Schoch A.B., Carleen S.A., Matsumoto D. (2017). An analysis of the role of nonreactive plasticizers in the crosslinking reactions of a rigid resin. J. Polym. Sci. B Polym. Phys..

[41] Stephenson C.R.J., Yoon T.P., MacMillan D.W.C. (2017). Visible Light Photocatalysis in Organic Chemistry.

[42] Konig B. (2017). Photocatalysis in Organic Synthesis—Past, Present, and Future. Eur. J. Org. Chem..

[43] Prier C.K., Rankic D.A., MacMillan D.W. (2013). Visible Light Photoredox Catalysis with Transition Metal Complexes: Applications in Organic Synthesis. Chem. Rev..

[44] Dettori G., Gaspa S., Porcheddu A., De Luca L. (2014). A two-step tandem reaction to prepare hydroxamic acids directly from alcohols. Org. Biomol. Chem..

[45] Gaspa S., Carraro M., Pisano L., Porcheddu A., De Luca L. (2019). Trichloroisocianuric acid: A versatile and efficient chlorinationg and oxidizing reagent. Eur. J. Org. Chem..

[46] Tilstam U., Weinmann H. (2002). Trichloroisocyanuric acid: A safe and efficient oxidant. Org. Process Res. Dev..

[47] Protti S., Ravelli M., Fagnoni M., Albini A. (2009). Solar light-driven photocatalyzed alkylations. Chemistry on the window ledge. Chem. Commun..

[48] Zhang H., Muniz K. (2017). Selective piperidine synthesis exploiting iodine-catalyzed C_sp_^3^–H amination under visible light. ACS Catal..

[49] Saika A.J., Boran P., Phukan P. (2016). Use of bromine and bromo-organic compounds in organic synthesis. Chem. Rev..

[50] Beckwith A.L.J., Goodrich J.E. (1965). Free-radical rearrangement of N-chlor-anides: A synthesis of lactones. Aust. J. Chem..

[51] Veisi H. (2010). Direct oxidative conversion of alcohols, amines, aldehydes and benzyl halides into the corresponding nitriles with tricholroisocianuric acid in aqueous ammonia. Synthesis.

[52] Srilakshmi Krishanaveni N., Suredra K., Rama Rao K. (2004). A Simple and Highly Selective Biomimetic Oxidation of Alcohols and Epoxides with N-Bromosuccinimide in the Presence of β-Cyclodextrin in Water. Adv. Synth. Catal..

[53] Filler R. (1963). Oxidations and Dehydrogenations with N-Bromosuccinimide and Related N-Haloimides. Chem. Rev..

[54] Wilson S.R., Tofigh S., Misra R.N. (1982). A novel, nonoxidative method for the conversion of aldehydes to esters. J. Org. Chem..

[55] Zhao J., Sun H., Lu Y., Li J., Yu Z., Zhu H., Ma C., Meng Q., Peg X. (2022). A divergent photocatalysis strategy for selective aerobic oxidation of C(sp3)–H bonds promoted by disulfides. Green Chem..

[56] Chen P.-S., Chou C.-H. (1996). Pyrolytic Chemistry of Thenyl Benzoates. Tetrahedron.

[57] Ruso J.S., Rajendiran N., Kumaran R.S. (2014). Metal-free synthesis of aryl esters by coupling aryl carboxylic acids and aryl boroni cacids. Tetrahedron Lett..

[58] Gondo K., Oyamada J., Kitamura T. (2015). Palladium-Catalyzed Desilylative Acyloxylation of Silicon–CarbonBonds on (Trimethylsilyl)arenes: Synthesis of Phenol Derivativesfrom Trimethylsilylarenes. Org. Lett..

[59] Tu Y., Yuan L., Wang T., Wang C., Ke J., Zhao J. (2017). Palladium-catalyzed oxidative carbonylation of aryl hydrazines with CO and O_2_ at atmospheric pressure. J. Org. Chem..

[60] Ramanjaneyulu B.T., Reddy V., Arde P., Mahesh S., Anand R.V. (2013). Combining Oxidative N-Heterocyclic Carbene Catalysis with Click Chemistry: A Facile One-Pot Approach to 1, 2, 3-Triazole Derivatives. Chem. Asian J..

[61] Ullah E., McNulty J., Sliwinski M., Robertson A. (2012). One-step synthesis of reusable, polymer-supported tri-alkyl phosphine ligands. Application in Suzuki–Miyaura and alkoxycarbonylation reactions. Tetrahedron Lett..

[62] Dai M.-S., Zheng Z.-M., Zhang S.L. (2023). High-valent Cu (III)–CF 3 compound-mediated esterification reaction. Org. Biomol. Chem..

[63] Rout S.K., Guin S., Ghara K.K., Banerjee A., Patel B.K. (2012). Copper catalyzed oxidative esterification of aldehydes with alkylbenzenes via cross dehydrogenative coupling. Org. Lett..

[64] Liu H., Dong C., Zhang Z., Wu P., Jiang X. (2012). Transition-Metal-Free Aerobic Oxidative Cleavage of C–C Bonds in α-Hydroxy Ketones and Mechanistic Insight to the Reaction Pathway. Angew. Chem. Int. Ed..

[65] Liu H., Shi G., Pan S., Jiang Y., Zhang Y. (2013). Palladium-catalyzed benzylation of carboxylic acids with toluene via benzylic C–H activation. Org. Lett..

[66] Finney E.E., Ogawa K.A., Boydston A.J. (2012). Organocatalyzed anodic oxidation of aldehydes. J. Am. Chem. Soc..

[67] Taniguchi T., Hirose D., Ishibashi H. (2011). Esterification via iron-catalyzed activation of triphenylphosphine with air. ACS Catal..

[68] But T.Y.S., Lu J., Toy P.H. (2010). Organocatalytic Mitsunobu reactions with 3, 5-dinitrobenzoic acid. Synlett.

[69] Ackermann L., Gschrei C.J., Althammer A., Riederer M. (2006). Cross-coupling reactions of aryl and vinyl chlorides catalyzed by a palladium complex derived from an air-stable H-phosphonate. Chem. Comm..

[70] Inamoto K., Kuroda J.I., Hiroya K., Noda Y., Watanabe M., Sakamoto T. (2006). Synthesis and catalytic activity of a pincer-type bis (imidazolin-2-ylidene) nickel (II) complex. Organometallics.

[71] Tian Y., Ling A., Fang R., Tan R.X., Liu Z.Q. (2018). Free-radical anti-Markovnikov hydroalkylation of unactivated alkenes with simple alkanes. Green Chem..

[72] Chen X., Hu S., Chen R., Wang J., Wu M., Guo H., Sun S. (2018). Fe-catalyzed esterification of amides via C–N bond activation. RSC Adv..

[73] Hoffmann H.M.R., Haase K. (1981). The synthesis of acyl iodides. Synthesis.

