# Supplementary material for: Visible Light-Promoted Oxidative Cross-Coupling of Alcohols to Esters
Source: Molecules. 2024 Jan 23;29(3):570. doi: 10.3390/molecules29030570 (PMC10856673; doi:10.3390/molecules29030570)

Supporting Information

Visible light-promoted oxidative cross-coupling of alcohols to esters

Andrea Dellisanti [1], Elisa Chessa[1], Andrea Porcheddu[2], Massimo Carraro[1], Luisa Pisano[1], Lidia De Luca [1],* and Silvia Gaspa[1]

[1] Dipartimento di Scienze Chimiche, Fisiche, Matematiche e Naturali, Università degli Sudi di Sassari, Via Vienna 2, 07100 Sassari, Itlay
[2] Dipartimento di Scienze Chimiche e Geologiche, Università degli Sudi di Cagliari, 09042 Monserrato, Italy
* Correspondence: ldeluca@uniss.it

Table of contents

1. Experimental section ……………………………………………………………………………..S3

1.1. General information………………………………………………………… ……S3

1.2. General Procedure for the synthesis of methyl benzoate 5a under solar simulator irradiation …………………………………………………………………………………S3

1.3. General Procedure for the synthesis of methyl benzoate 5a under sunlight irradiation.S3

1.4. General Procedure to esters under blue LED irradiation ……………………………….S4

1.5. Compound characterizations ……………………………………………………….....S5

1.6. Detection and characterization of benzoyl chloride 3………………………………...S11

1.7. References…………………………………………………………………………..…S12

2. NMR Spectra of esters……………………………………………………………………..S14

2.1. NMR Spectra of Benzoyl chloride (3)……………………………………………………..S34

3. UV-Vis Spectra………………………………………………………………………...…S35

4. Experimental set-up………………………………………………………………………...S35

1. Experimental Section:

1.1. General Information:

All reagents and solvents were as obtained by commercial source. All the reactions were run under Argon atmosphere using standard techniques. All solvents were dried by usual methods and distilled under Argon. Column chromatography was generally performed on silica gel (pore size 60 Å, 32-63 nm particle size) and reactions were monitored by thin-layer chromatography (TLC) analysis was performed with Merck Kieselgel 60 F254 plates and visualized using UV light at 254 nm, $KMnO_4$, 2,4-DNP and cerium ammonium molybdate staining. The reactions were conducted with Abet tech sun 2000 simulator (under 100 mW/cm2 simulated AM 1.5G irradiance). For irradiation with blue light OSRAM Oslon SSL 80 LDCQ7P-1U3U (blue, λ max = 455 nm, I max = 1000 mA, 1.12 W) was used. ^{1}H NMR and ^{13}C NMR spectra were measured on a Bruker Avance III 400 spectrometer (400 MHz or 100 MHz, respectively) using $CDCl_3$ solutions and TMS as an internal standard. Chemical shifts are reported in parts per million (ppm, d) relative to internal tetramethylsilane standard (TMS, d 0.00). The peak patterns are indicated as follows: s, singlet; d, doublet; t, triplet; m, multiplet; q, quartet; dd, doublet of doublets; br, broad. The coupling constants, J, are reported in Hertz (Hz). High resolution mass spectra HRMS (HESI-FT-ORBITRAP) were recorded on a Q-Exactive Thermo Scientific mass spectrometer. Melting points were determined in open capillary tubes and are uncorrected.

1.2. General Procedure for the synthesis of methyl benzoate 5a under solar simulator irradiation:

In a round bottom flask of 10 mL, equipped with a condenser, TCCA (1.3 mmol) was added to a solution of a benzyl alcohol (1.1 mmol) in 2 mL dichloromethane under Ar atmosphere and at room temperature. The resulting suspension was stirred under solar simulator irradiation for 1.5 hours under Ar (the reaction was monitored by TLC until disappearance of benzyl alcohol). Then the reaction mixture was cooled to 0 °C, stirred under an inert atmosphere of dry argon and methanol (1.0 mmol) was dropwise added via syringe followed by dropwise addition of NEt_3 (2.0 mmol) and then DMAP (10% mol) at once. After completion of the addition, the reaction mixture left to stir at room temperature until disappearance of the methanol (monitored by TLC, the reaction is usually complete in about 1 h). Then the solvent was removed under vacuum, and the residue purified by flash chromatography. Pale yellow oil; (0.135 g, 99 % yield).

1.3. General Procedure for the synthesis of methyl benzoate 5a under sunlight irradiation:

In a round bottom flask of 10 mL, equipped with a condenser, TCCA (1.3 mmol) was added to a solution of a benzyl alcohol (1.1 mmol) in 2 mL dichloromethane under Ar atmosphere and at room temperature. The resulting suspension was stirred under sun light irradiation for 1.5 hours under Ar (the reaction was monitored by TLC until disappearance of benzyl alcohol). Then the reaction mixture was cooled to 0 °C, stirred under an inert atmosphere of dry argon and methanol (1.0 mmol) was dropwise added via syringe followed by dropwise

addition of NEt_3 (2.0 mmol) and then DMAP (10% mol) at once. After completion of the addition, the reaction mixture left to stir at room temperature until disappearance of the methanol (monitored by TLC, the reaction is usually complete in about 1 h). Then the solvent was removed under vacuum, and the residue purified by flash chromatography. Pale yellow oil; (0.134 g, 98 % yield).

1.4. General Procedure to esters under blue LED irradiation:

In a round bottom flask of 10 mL, equipped with a condenser, TCCA (1.1 mmol) was added to a solution of a benzyl alcohol (1.1 mmol) in 1 mL dichloromethane under Ar atmosphere and at room temperature. The resulting suspension was stirred and irradiated using a blue LED (455 nm) for 1.5 h at 25 °C under Ar (the reaction was monitored by TLC until disappearance of benzyl alcohol). Then the reaction mixture was cooled to 0 °C, stirred under an inert atmosphere of dry argon and an alcohol (1.0 mmol) was dropwise added via syringe followed by dropwise addition of NEt_3 (2.0 mmol) and then DMAP (10% mol) at once. After completion of the addition, the reaction mixture left to stir at room temperature until disappearance of the alcohol (monitored by TLC, the reaction is usually complete in about 1 h). Then the solvent was removed under vacuum, and the residue purified by flash chromatography.

1.5. Compound characterizations:

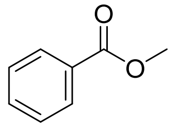

Methyl benzoate (5a):[1] Pale yellow oil; (0.135 g, 99 % yield); R_f= 0.484 (hexane/ethyl acetate 4.7/0.3). ^{1}H NMR (400 MHz, $CDCl_3$) δ: 8.03 (d, J = 7.6 Hz, 2H), 7.54 (t, J = 7.4 Hz, 1H), 7.42 (t, J = 7.7 Hz, 2H), 3.90 (s, 3H). ^{13}C NMR (100 MHz, $CDCl_3$) δ: 166.9, 132.8, 130.1, 129.4, 128.2, 51.9.

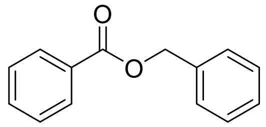

Benzyl benzoate (5b):[2] Colorless oil; (0.182 g, 86 % yield); R_f = 0.448 (hexane/ethyl acetate 4.5:0.5). ^{1}H NMR (400 MHz, $CDCl_3$) δ: 8.07 (d, J = 7.8 Hz, 2H), 7.53 (t, J = 7.3 Hz, 1H), 7.49 – 7.31 (m, 7H), 5.36 (s, 2H). ^{13}C NMR (100 MHz, $CDCl_3$) δ: 166.5, 136.1, 133.1, 130.2, 129.8, 128.6, 128.4, 128.3, 128.2, 66.7.

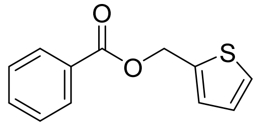

Thiophen-2-ylmethyl benzoate (5c): Colorless oil; (0.203 g, 93 % yield); R_f= 0.36 (hexane/ethyl acetate 4.5/0.5). ^{1}H NMR (400 MHz, $CDCl_3$) δ : 8.04 (d, J = 7.9 Hz, 2H), 7.49 (t, J = 7.3 Hz, 1H), 7.37 (t, J = 7.4 Hz, 2H), 7.28 (d, J = 5.0 Hz, 1H), 7.13 (m, 1H), 6.97 - 6.95 (m, 1H), 5.48 (s, 2H).[3] ^{13}C NMR (100 MHz, $CDCl_3$) δ : 166.0, 137.9, 132.9, 129.8, 129.6, 128.2, 128.0, 126.7, 126.6, 60.8.[3]

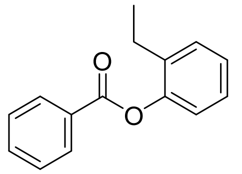

2-Ethylphenyl benzoate (5d): Colorless oil; (0.186 g, 82 % yield); R_f= 0.44 (hexane/ethyl acetate 4.7/0.3).

^{1}H NMR (400 MHz, $CDCl_3$) δ : 8.23 - 8.21 (m, 2H), 7.62 - 7.46 (m, 1H), 7.50 - 7.46 (m, 2H), 7.30 – 7.13 (m, 4H), 2.61 (q, *J* = 7.5 Hz, 2H), 1.20 (t, *J* = 7.5 Hz, 3H).[4] ^{13}C NMR (100 MHz, $CDCl_3$) δ : 165.2, 149.2, 136.1, 133.6, 130.2, 129.7, 129.6, 128.7, 127.0, 126.3, 122.4, 23.4, 14.4.[4]

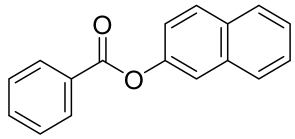

Naphthalen-2-yl benzoate (5e): White solid; (0.201 g, 81 % yield); m.p= 101-103 °C;[5] R_f = 0.492 (hexane/ethyl acetate 4.5/0.5). ^{1}H NMR (400 MHz, $CDCl_3$) δ: 8.29 (d, *J* = 7.7 Hz, 2H), 7.95 – 7.85 (m, 3H), 7.73 (d, *J* = 2.3 Hz, 1H), 7.67 (t, *J* = 7.4 Hz, 1H), 7.58 – 7.49 (m, 4H), 7.40 (dd, *J* = 8.9, 2.3 Hz, 1H).[6] ^{13}C NMR (100 MHz, $CDCl_3$) δ: 165.3, 148.6, 133.8, 133.6, 131.5, 130.2, 129.5, 129.4, 128.6, 127.8, 127.6, 126.5, 125.7, 121.2, 118.7.[6]

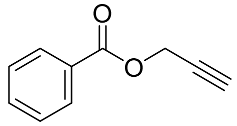

Prop-2-yn-1-yl benzoate (5f):[7] Colorless oil; (0.114 g, 71 % yield); R_f= 0.46 (hexane/ethyl acetate 4.5/0.5). ^{1}H NMR (400 MHz, $CDCl_3$) δ : 8.08 - 8.06 (m, 2H), 7.60 - 7.56 (m, 1H), 7.47 - 7.43 (m, 2H), 4.94 (d, *J* = 2.4 Hz, 2H), 2.52 (t, *J* = 2.4 Hz, 1H). ^{13}C NMR (100 MHz, $CDCl_3$) δ : 165.8, 133.3, 129.8, 129.4, 128.5, 77.7, 75.0, 52.4.

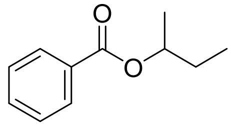

***Sec*-butyl benzoate (5g):**[8] Yellow oil; (0.123 g, 69 % yield); R_f= 0.55 (hexane/ethyl acetate 4.5/0.5). ^{1}H NMR (400 MHz, $CDCl_3$) δ : 8.05 (d, *J* = 8.0 Hz, 2H), 7.55 (t, *J* = 7.1 Hz, 1H), 7.43 (t, *J* = 7.6 Hz, 2H), 5.10 (m, 1H), 1.71 (m, 2H), 1.34 (d, *J* = 6.2 Hz, 3H), 0.98 (t, *J* = 7.4 Hz, 3H). ^{13}C NMR (100 MHz, $CDCl_3$) δ: 166.3, 132.7, 130.9, 129.5, 128.3, 72.8, 29.0, 19.6, 9.7.

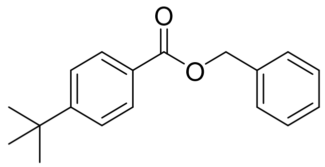

Benzyl 4-(*tert*-butyl)benzoate (6a):[9] Colorless oil; (0.215 g, 80 % yield); R_f = 0.38 (hexane/ethyl acetate 4.7/0.3). ^{1}H NMR (400 MHz, $CDCl_3$) δ: 8.01 (d, J = 8.1 Hz, 2H), 7.45 - 7.43 (m, 4H), 7.39 – 7.32 (m, 3H), 5.35 (s, 2H), 1.33 (s, 9H). ^{13}C NMR (100 MHz, $CDCl_3$) δ: 166.5, 156.7, 136.3, 129.6, 128.6, 128.2, 128.1, 127.4, 125.4, 66.5, 35.1, 31.1.

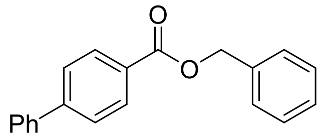

Benzyl [1,1'-biphenyl]-4-carboxylate (6b):[10] White solid; (0.260 g, 90 % yield); m.p. 115 - 117 °C, R_f = 0.41 (hexane/ethyl acetate 4.5/0.5). ^{1}H NMR (400 MHz, $CDCl_3$) δ: 8.17 (d, J = 8.4 Hz, 2H), 7.69 - 7.63 (m, 4H), 7.52 – 7.37 (m, 8H), 5.42 (s, 2H). ^{13}C NMR (100 MHz, $CDCl_3$) δ: 166.3, 145.7, 139.9, 136.1, 130.2, 128.9, 128.8, 128.6, 128.2, 128.1, 128.1, 127.2, 127.0, 66.7.

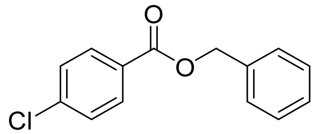

Benzyl 4-chlorobenzoate (6c):[11] Yellow oil; (0.237 g, 96 % yield); R_f = 0.48 (hexane/ethyl acetate 4.5/0.5). ^{1}H NMR (400 MHz, $CDCl_3$) δ: 8.04 – 7.99 (m, 2H), 7.46 – 7.34 (m, 7H), 5.36 (s, 2H). ^{13}C NMR (100 MHz, $CDCl_3$) δ: 165.6, 139.5, 135.8, 131.1, 128.7, 128.6, 128.6, 128.4, 128.2, 66.9.

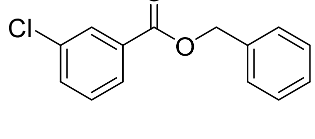

Benzyl 3-chlorobenzoate (6d):[12] Yellow oil; (0.232 g, 94 % yield); R_f = 0.42 (hexane/ethyl acetate 4./0.5). ^{1}H NMR (400 MHz, $CDCl_3$) δ: 8.05 (t, J = 1.9 Hz, 1H), 7.96 (dt, J = 7.8, 1.4 Hz, 1H), 7.55 – 7.53 (m, 1H), 7.47 – 7.35 (m, 6H), 5.37 (s, 2H). ^{13}C NMR (100 MHz, $CDCl_3$) δ: 165.2, 135.6, 134.5, 133.0, 131.9, 129.7,

129.7, 128.6, 128.4, 128.3, 127.8, 67.1.

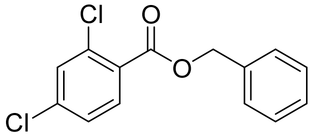

Benzyl 2,4-dichlorobenzoate (6e):[13] Yellow oil; (0.239 g, 85 % yield); R_f = 0.48 (hexane/ethyl acetate 4.4/0.6). ^{1}H NMR (400 MHz, $CDCl_3$) δ: 7.83 (d, J = 8.4 Hz, 1H), 7.49 – 7.35 (m, 6H), 7.29 (dd, J = 8.5, 2.0 Hz, 1H), 5.37 (s, 2H). ^{13}C NMR (100 MHz, $CDCl_3$) δ: 164.5, 138.4, 135.4, 135.1, 132.6, 131.1, 128.6, 128.5, 128.4, 128.2, 127.0, 67.5.

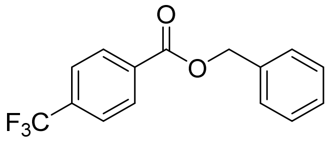

Benzyl 4-(trifluoromethyl)benzoate (6f):[12] Yellow oil; (0.098 g, 35 % yield); R_f= 0.46 (hexane/ethyl acetate 4.8/0.2). ^{1}H NMR (400 MHz, $CDCl_3$) δ: 8.20 (d, J = 7.8 Hz, 2H), 7.71 (d, J = 8.2 Hz, 2H), 7.48 – 7.35 (m, 5H), 5.41 (s, 2H). ^{13}C NMR (100 MHz, $CDCl_3$) δ: 165.2, 135.6, 134.5 (d, JC-F = 32.4 Hz), 133.3 (d, $J_{C\text{-}F}$ = 0.9 Hz), 130.1, 128.7, 128.5, 128.3, 125.4 (q, $J_{C\text{-}F}$ = 3.7 Hz), 123.6 (d, $J_{C\text{-}F}$ = 271.6 Hz), 67.2.

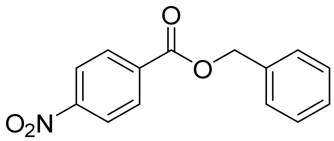

Benzyl 4-nitrobenzoate (6g): Yellow solid; (0.057 g, 22 % yield); R_f= 0.516 (hexane/ethyl acetate 4.2/0.8); m.p.= 82-83°C .[14] ^{1}H NMR (400 MHz, $CDCl_3$) δ: 8.29 – 8.22 (m, 4H), 7.47 (d, J = 7.2 Hz, 2H), 7.44 – 7.36 (m, 3H), 5.41 (s, 2H).[15] ^{13}C NMR (100 MHz, $CDCl_3$) δ: 164.4, 150.5, 135.4, 135.2, 130.7, 128.6, 128.5, 128.3, 123.4, 67.5.[15]

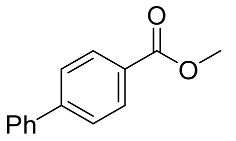

Methyl [1,1'-biphenyl]-4-carboxylate (7a): White solid; (0.180 g, 85 % yield); m.p= 116-117 °C;[16] R_f= 0.303 (hexane/ethyl acetate 4.8/0.2). ^{1}H NMR (400 MHz, $CDCl_3$) δ: 8.12 (d, J = 8.3 Hz, 2H), 7.65 (dd, J = 14.7, 7.9 Hz, 4H), 7.47 (t, J = 7.5 Hz, 2H), 7.40 (t, J = 7.3 Hz, 1H), 3.95 (s, 3H).[17] ^{13}C NMR (100 MHz, $CDCl_3$) δ: 166.9, 145.5, 139.9, 130.0, 128.9, 128.8, 128.1, 127.2, 126.9, 52.0.[17]

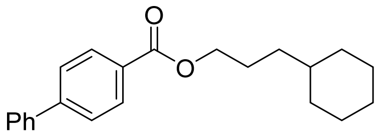

3-cyclohexylpropyl [1,1'-biphenyl]-4-carboxylate (7b):[18] Pale yellow oil; (0.268 g, 83 % yield); R_f= 0.486 (hexane/ethyl acetate 4.8/0.2). ^{1}H NMR (400 MHz, $CDCl_3$) δ: 8.13 (d, J = 8.2 Hz, 2H), 7.65 (dd, J = 14.7, 7.9 Hz, 4H), 7.48 (t, J = 7.5 Hz, 2H), 7.40 (t, J = 7.3 Hz, 1H), 4.34 (t, J = 6.8 Hz, 2H), 1.84 – 1.66 (m, 7H), 1.37 – 1.17 (m, 6H), 0.98 – 0.89 (m, 2H). ^{13}C NMR (100 MHz, $CDCl_3$) δ: 166.5, 145.5, 140.0, 130.0, 129.3, 128.9, 128.0, 127.2, 126.9, 65.4, 37.3, 33.7, 33.3, 26.6, 26.3, 26.1.

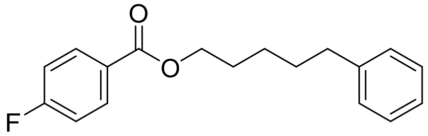

5-phenylpentyl 4-fluorobenzoate (7c): Colorless oil; (0.244 g, 85 % yield); R_f= 0.515 (hexane/ethyl acetate 4.7/0.3). ^{1}H NMR (400 MHz, $CDCl_3$) δ: 8.07 (dd, J = 8.7, 5.5 Hz, 2H), 7.35 – 7.28 (m, 2H), 7.25 – 7.19 (m, 3H), 7.12 (t, J = 8.6 Hz, 2H), 4.34 (t, J = 6.6 Hz, 2H), 2.68 (t, J = 7.7 Hz, 2H), 1.83 (dt, J = 14.4, 6.8 Hz, 2H), 1.79 – 1.71 (m, 2H), 1.53 (q, J = 8.1 Hz, 2H). ^{13}C NMR (100 MHz, $CDCl_3$) δ: 165.6 (d, $J_{C\text{-}F}$ = 252 Hz), 165.5, 142.22, 132.0 (d, $J_{C\text{-}F}$ = 9 Hz), 128.29, 128.19, 126.6 (d, $J_{C\text{-}F}$ = 3 Hz), 125.63, 115.3 (d, $J_{C\text{-}F}$ = 22 Hz), 64.96, 35.64, 30.92, 28.46, 25.50. HRMS (HESI-FT-ORBITRAP) calcd for $C_{18}H_{19}FNaO_2$ [M+Na]$^+$: 309,1267, found 309,1269.

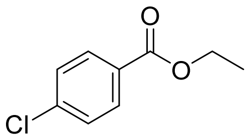

Ethyl 4-chlorobenzoate (7d):[19] Colorless oil; (0.148 g, 80 % yield); R_f= 0.49 (hexane/ethyl acetate 4.8/0.2).

^{1}H NMR (400 MHz, $CDCl_3$) δ: 7.95 (d, *J* = 8.5 Hz, 2H), 7.38 (d, *J* = 8.5 Hz, 2H), 4.35 (q, *J* = 7.1 Hz, 2H), 1.37 (t, *J* = 7.2 Hz, 3H). ^{13}C NMR (100 MHz, $CDCl_3$) δ: 165.6, 139.1, 130.8, 128.9, 128.6, 61.1, 14.2.

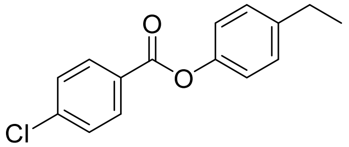

4-ethylphenyl 4-chlorobenzoate (7e): Colorless oil; (0.258 g, 99 % yield); R_f= 0.486 (hexane/ethyl acetate 4.5/0.5). ^{1}H NMR (400 MHz, $CDCl_3$) δ: 8.22 (d, *J* = 8.5 Hz, 2H), 7.54 (d, *J* = 8.5 Hz, 2H), 7.39 – 7.37 (m, 1H), 7.35 – 7.27 (m, 2H), 7.23 – 7.20 (m, 1H), 2.68 (q, *J* = 7.6 Hz, 2H), 1.28 (t, *J* = 7.6 Hz, 3H). ^{13}C NMR (100 MHz, $CDCl_3$) δ: 164.1, 148.8, 140.0, 135.8, 131.47, 129.4, 128.9, 127.9, 126.9, 126.3, 122.1, 23.2, 14.1. HRMS (HESI-FT-ORBITRAP) calcd for $C_{15}H_{13}ClNaO_2$ $[M+Na]^+$: 283,0502, found 283,0500.

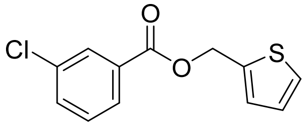

Thiophen-2-ylmethyl 3-chlorobenzoate (7f): Colorless oil; (0.223 g, 88 % yield); R_f= 0.469 (hexane/ethyl acetate 4.7/0.3). ^{1}H NMR (400 MHz, $CDCl_3$) δ: 8.03 (s, 1H), 7.94 (d, *J* = 7.8 Hz, 1H), 7.52 (d, *J* = 8.6 Hz, 1H), 7.36 (t, *J* = 6.9 Hz, 2H), 7.19 (d, *J* = 3.4 Hz, 1H), 7.07 – 6.96 (m, 1H), 5.52 (s, 2H). ^{13}C NMR (100 MHz, $CDCl_3$) δ: 164.9, 137.5, 134.4, 133.0, 131.6, 129.7, 129.6, 128.4, 127.8, 127.0, 126.8, 61.2. HRMS (HESI-FT-ORBITRAP) calcd for $C_{12}H_9ClNaO_2S$ $[M+Na]^+$: 274,9910, found 274,9912.

1.6. Detection and characterization of benzoyl chloride (3):

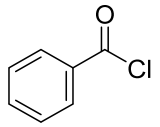

In a round bottom flask of 10 mL, equipped with a condenser, TCCA (2.2 mmol) was added to a solution of benzyl alcohol (2.2 mmol) in 2 mL dichloromethane under Ar atmosphere and at room temperature. The resulting suspension was stirred under blue LED irradiation (455 nm) for 1.5 hours under Ar (the reaction was monitored by TLC until disappearance of benzyl alcohol). The mixture of reaction was filtered on Celite, then the solvent was removed under vacuum and the residue was distilled.^{1}H NMR (400 MHz, CDCl3) δ: 8.08 (d, J = 7.8 Hz, 2H), 7.66 (t, J = 7.4 Hz, 1H), 7.48 (t, J = 7.6 Hz, 2H). ^{13}C NMR (100 MHz, CDCl3) δ: 168.3, 135.4, 133.2, 131.4, 129.0.[20]

1.7. References:

[1] Zhao, J.; Sun, H.; Lu, Y.; Li, J.; Yu, Z.; Zhu, H.; Ma, C.; Meng, Q.; Peg, X. A divergent photocatalysis strategy for selective aerobic oxidation of C(sp^3)–H bonds promoted by disulfides. *Green Chem.* **2022**, *24*, 8503-8511.

[2] Jie, F.; Shuai, L.; Shan-Yong, C.; Ji, Z.; Song-Sen, F.; Xiao-Qi, Y. A Metal-Free Oxidative Esterification of the Benzyl C-H Bond. *Adv. Synth. & Catal.* **2012**, *354*, 1287-1292.

[3] Chen, P.-S.; Chou, C.-H. Pyrolytic Chemistry of Thenyl Benzoates. *Tetrahedron* **1996**, *52*, 13615-13622.

[4] Ruso, J. S.; Rajendiran, N.; Kumaran, R. S. Metal-free synthesis of aryl esters by coupling aryl carboxylic acids and aryl boroni cacids. *Tetrahedron Lett.* **2014**, *55*, 2345-2347.

[5] Gondo, K.; Oyamada, J.; Kitamura, T. Palladium-Catalyzed Desilylative Acyloxylation of Silicon−CarbonBonds on (Trimethylsilyl)arenes: Synthesis of Phenol Derivativesfrom Trimethylsilylarenes. *Org. Lett.* **2015**, *17*, 4778-4781.

[6] Tu, Y.; Yuan, L.; Wang, T.; Wang, C.; Ke, J.; Zhao, J. Palladium-catalyzed oxidative carbonylation of aryl hydrazines with CO and O2 at atmospheric pressure. *J. Org. Chem.* **2017**, *82*, 4970-4976.

[7] Ramanjaneyulu, B. T.; Reddy, V.; Arde, P.; Mahesh, S.; Anand, R. V. Combining Oxidative N-Heterocyclic Carbene Catalysis with Click Chemistry: A Facile One-Pot Approach to 1, 2, 3-Triazole Derivatives. *Chem. Asian J.* **2013**, *8*, 1489-1496.

[8] Ullah, E.; McNulty, J.; Sliwinski, M.; Robertson, A. One-step synthesis of reusable, polymer-supported tri-alkyl phosphine ligands. Application in Suzuki–Miyaura and alkoxycarbonylation reactions. *Tetrahedron Lett.* **2012**, *53*, 3990-3993.

[9] Dai, M.-S.; Zheng, Z.-M.; Zhang, S. L. High-valent Cu (III)–CF 3 compound-mediated esterification reaction. *Org. Biomol.Chem.* **2023**, *21*, 935-939.

[10] Rout, S. K.; Guin, S.; Ghara, K. K.; Banerjee, A.; Patel, B. K. Copper catalyzed oxidative esterification of aldehydes with alkylbenzenes via cross dehydrogenative coupling. *Org. Lett.* **2012**, *14*, 3982-3985.

[11] Liu, H.; Dong, C.; Zhang, Z.; Wu, P.; Jiang, X. Transition-Metal-Free Aerobic Oxidative Cleavage of C–C Bonds in α-Hydroxy Ketones and Mechanistic Insight to the Reaction Pathway. *Angew Chem.Int. Ed.* **2012**, *51*, 12570-12574.

[12] Liu, H.; Shi, G.; Pan, S.; Jiang, Y.; Zhang, Y. Palladium-catalyzed benzylation of carboxylic acids with toluene via benzylic C–H activation. Org. Lett. **2013**, *15*, 4098-4101.

[13] Finney, E. E.; Ogawa, K. A.; Boydston, A. J. Organocatalyzed anodic oxidation of aldehydes. *J. Am. Chem. Soc.* **2012**, *134*, 12374-12377.

[14] Taniguchi, T.; Hirose, D.; Ishibashi, H. Esterification via iron-catalyzed activation of triphenylphosphine with air. *ACS Catal.* **2011**, *1*, 1469-1474.

[15] But, T. Y. S.; Lu, J.; Toy, P. H. Organocatalytic Mitsunobu reactions with 3, 5-dinitrobenzoic acid. *Synlett* **2010**, *2010*, 1115-1117.

[16] Ackermann, L.; Gschrei, C. J.; Althammer, A.; Riederer, M. Cross-coupling reactions of aryl and vinyl chlorides catalyzed by a palladium complex derived from an air-stable H-phosphonate. *Chem. Comm.* **2006**, 1419-1421.

[17] Inamoto, K.; Kuroda, J. I.; Hiroya, K.; Noda, Y.; Watanabe, M.;Sakamoto, T. Synthesis and catalytic activity of a pincer-type bis (imidazolin-2-ylidene) nickel (II) complex. *Organometallics* **2006**, *25*, 3095-3098.

[18] Tian, Y.; Ling, A.; Fang, R.; Tan, R. X.;Liu, Z. Q. Free-radical anti-Markovnikov hydroalkylation of unactivated alkenes with simple alkanes. *Green Chem.* **2018**, *20*, 3432-3435.

[19] Chen, X.; Hu, S.; Chen, R.; Wang, J.; Wu, M.; Guo, H.; Sun, S. Fe-catalyzed esterification of amides via C–N bond activation. *RSC Adv.* **2018**, *8*, 4571-4576.

[20] Hoffmann, H. M. R.; Haase, K. The synthesis of acyl iodides. *Synthesis* **1981**, *1981*, 715-719

2. NMR Spectra of esters:

Methyl benzoate (5a):

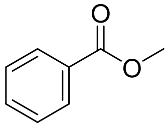

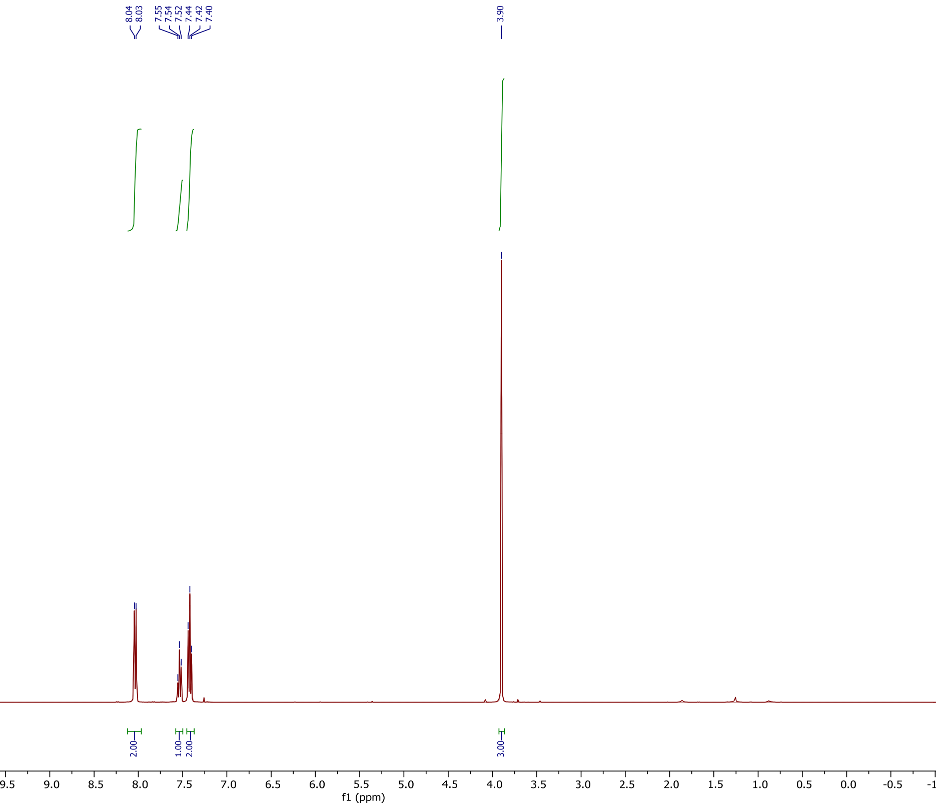

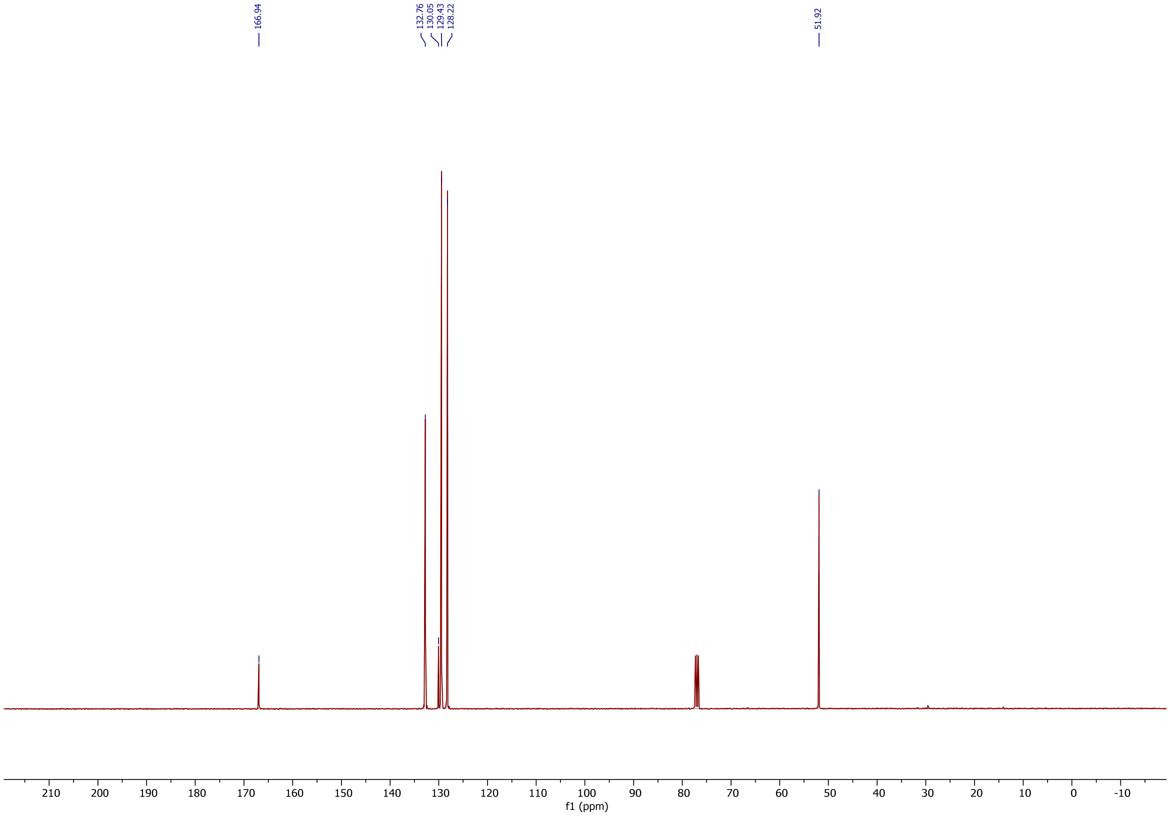

Benzyl benzoate (5b):

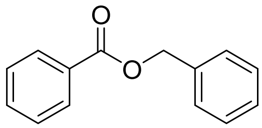

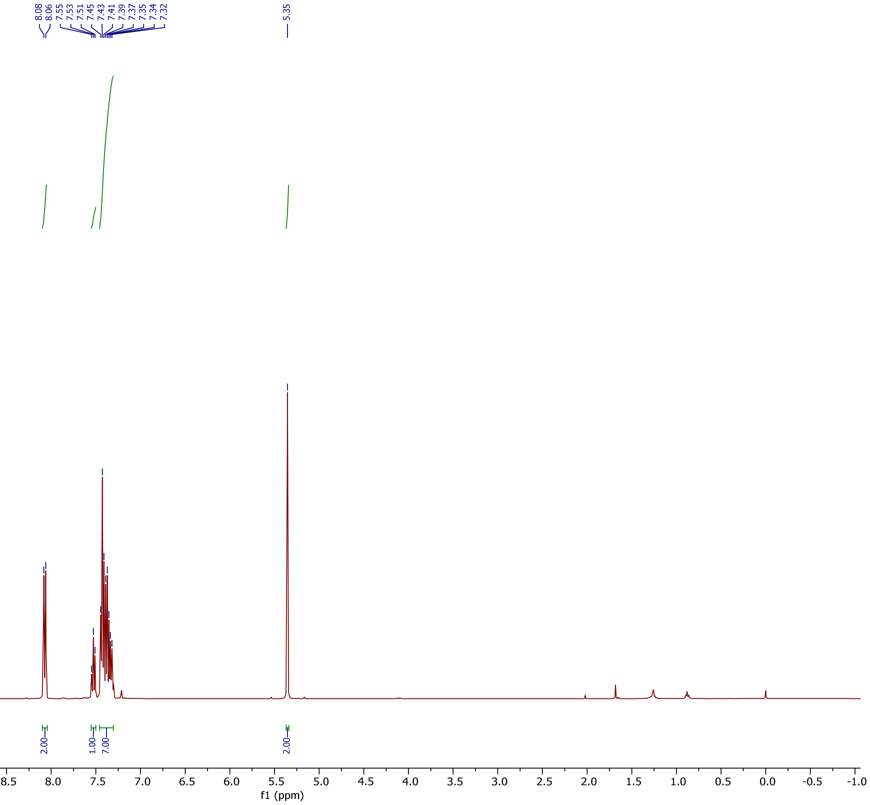

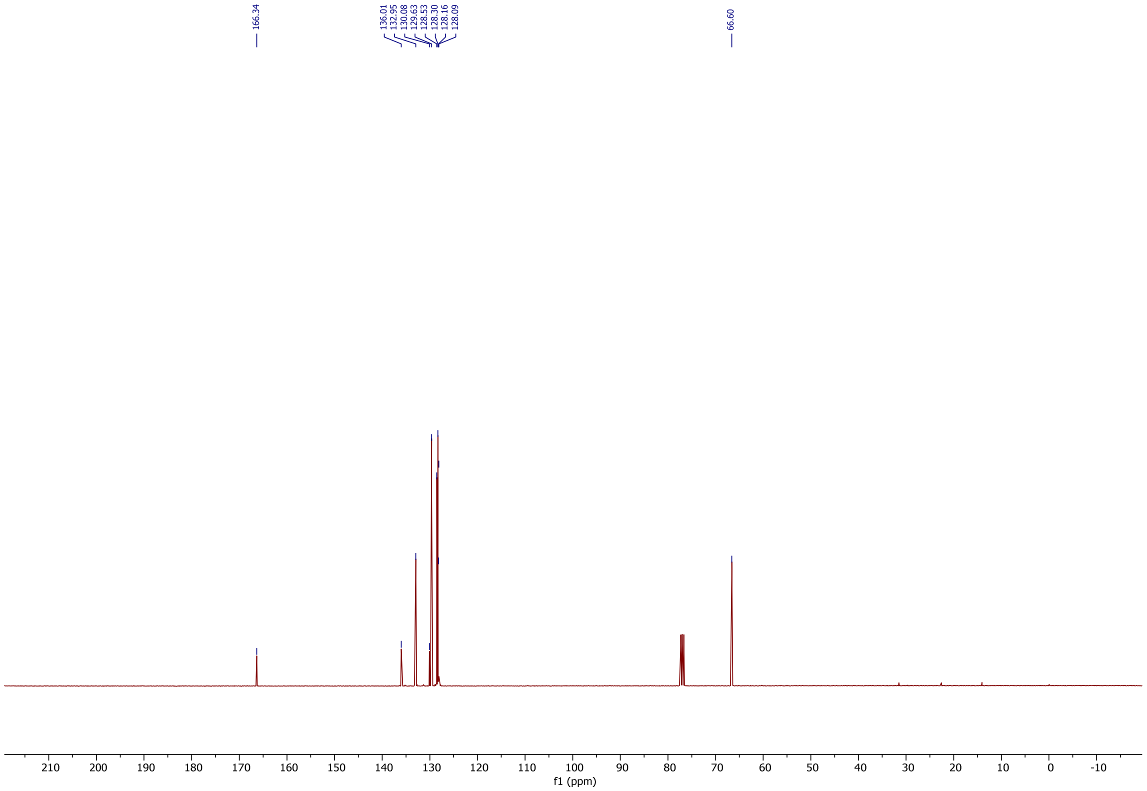

Thiophen-2-ylmethyl benzoate (5c):

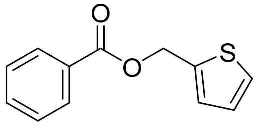

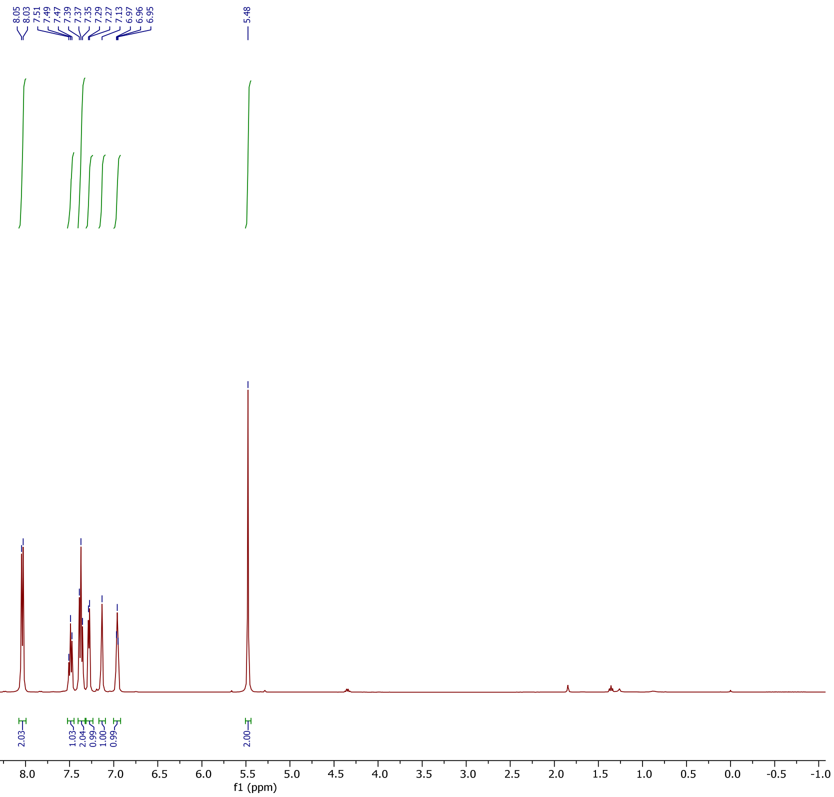

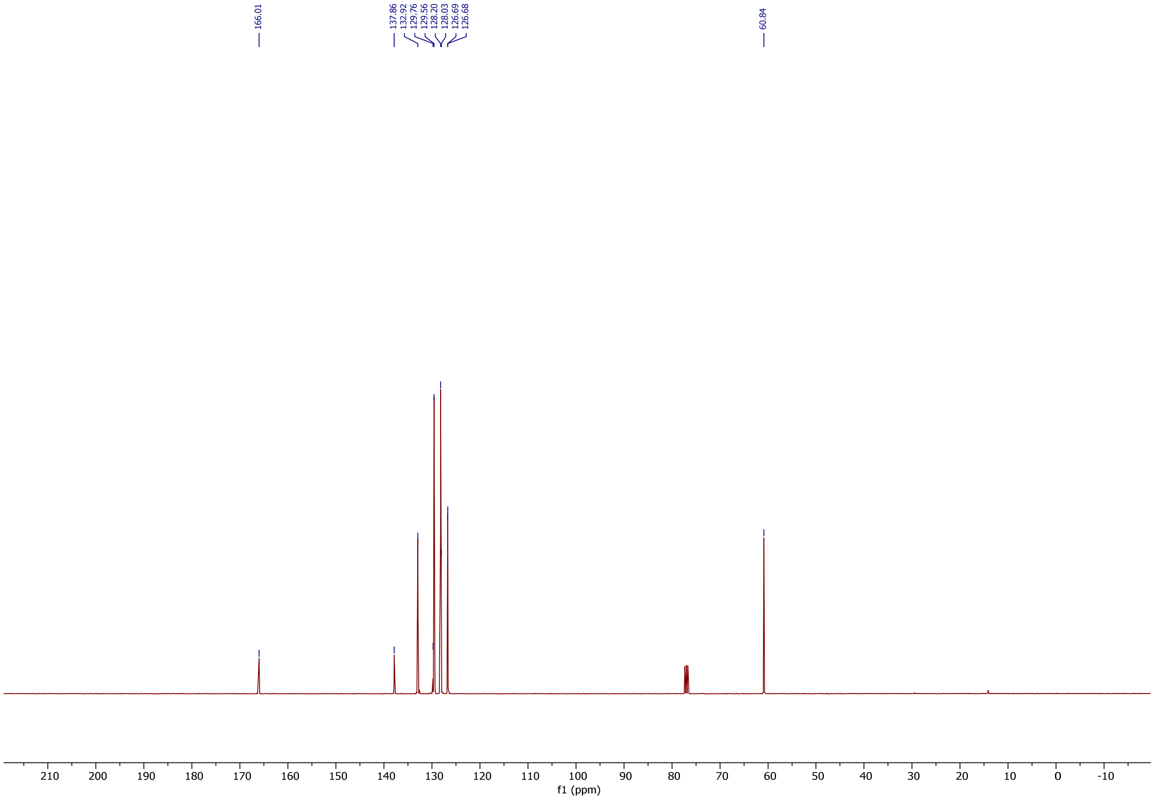

2-Ethylphenyl benzoate (5d):

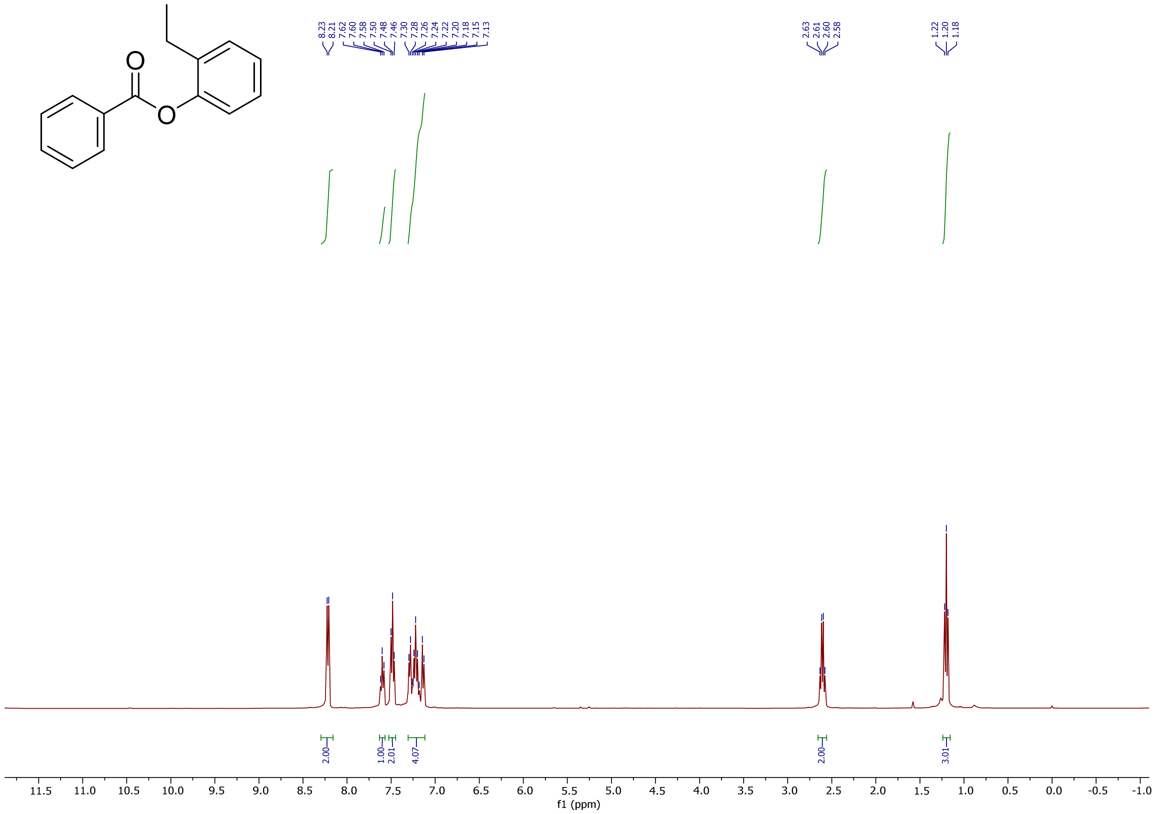

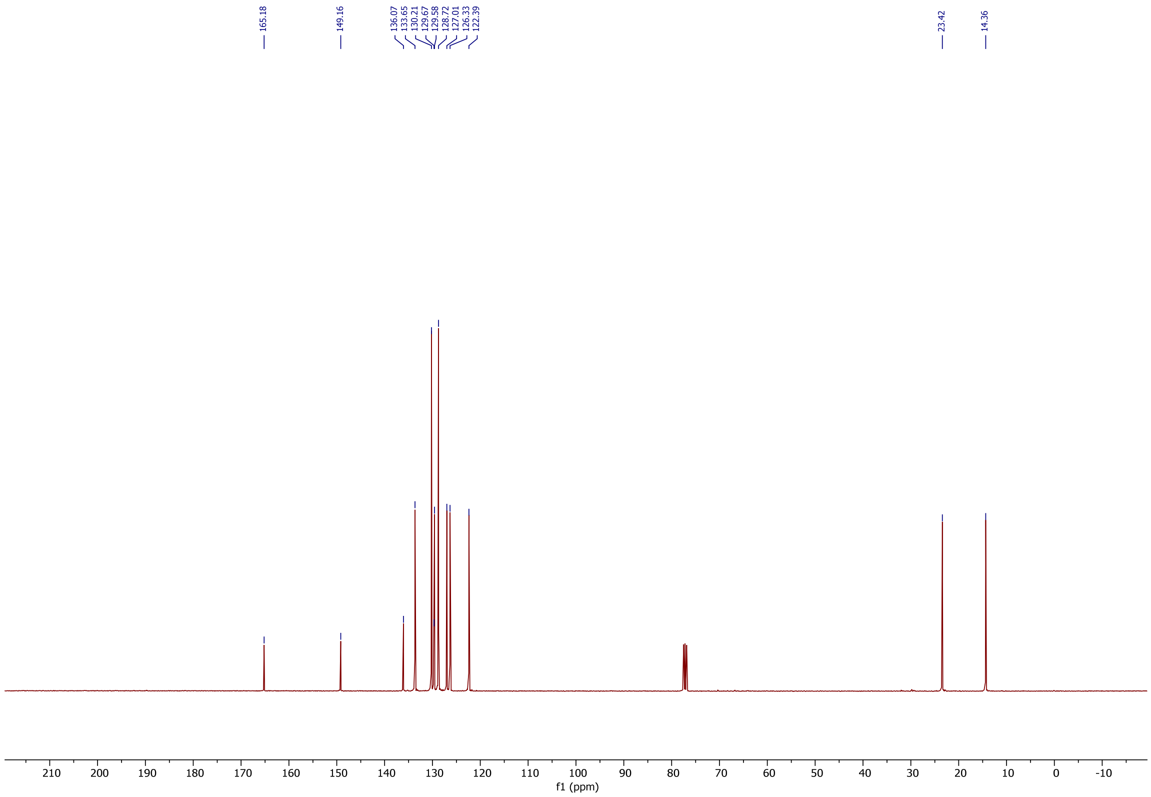

Naphthalen-2-yl benzoate (5e):

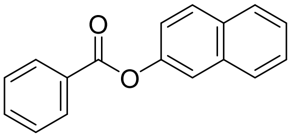

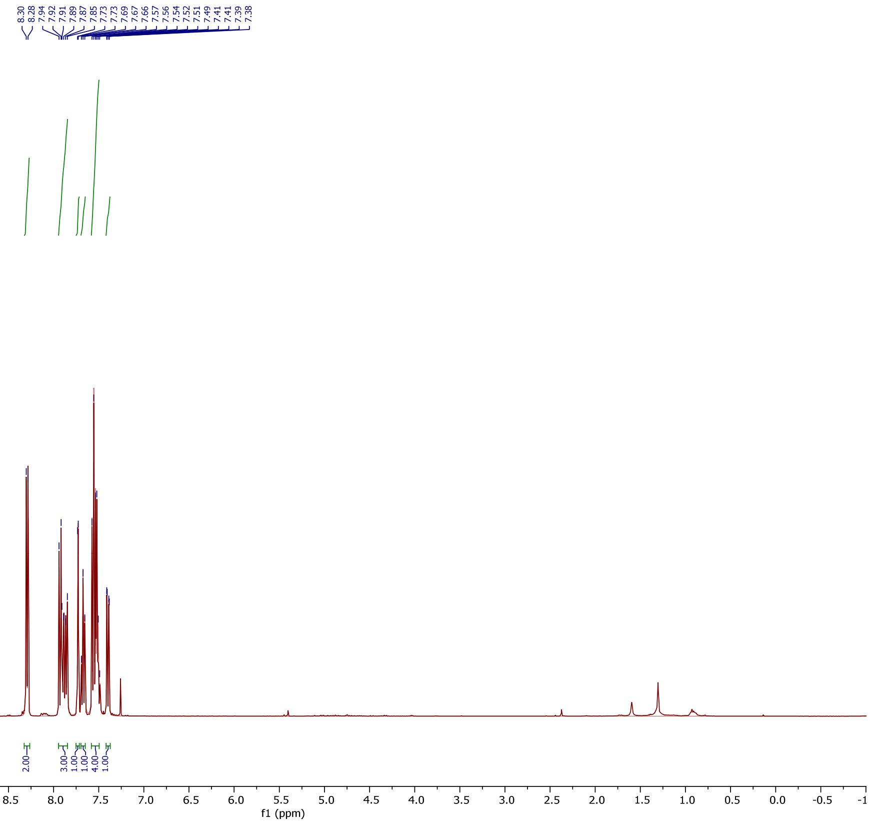

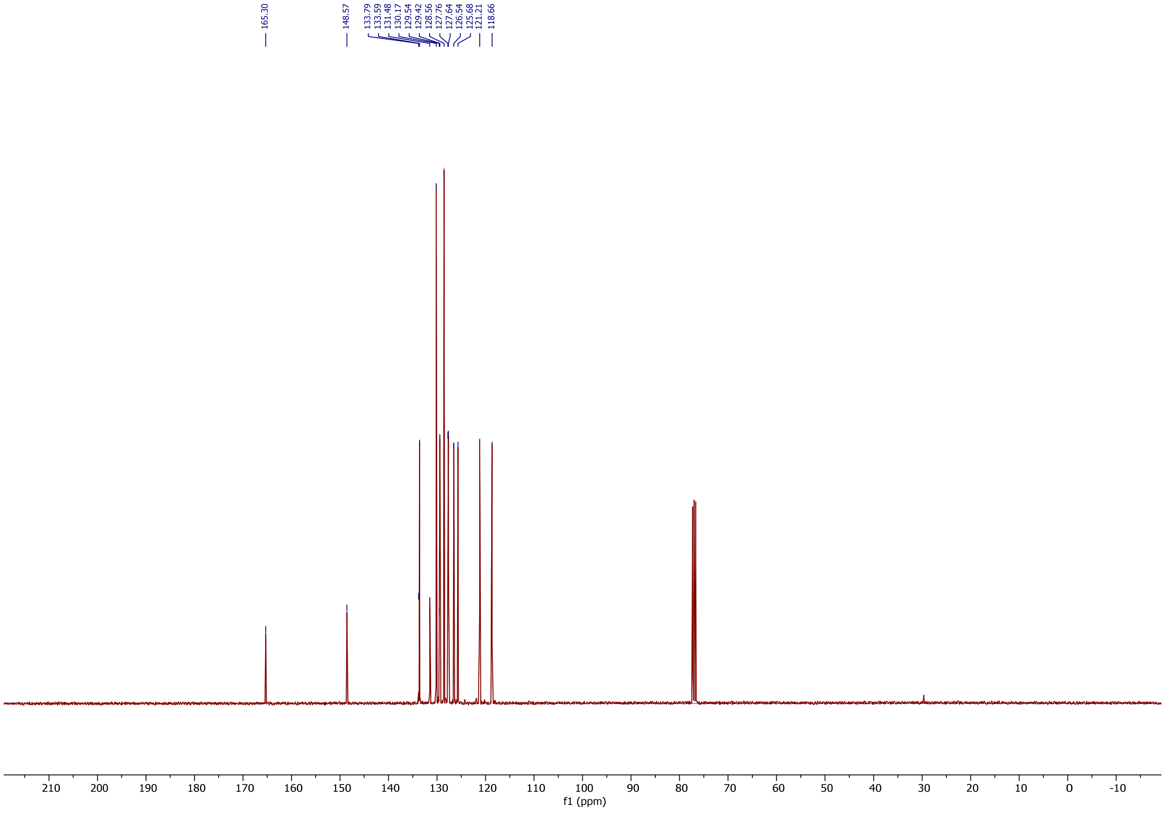

Prop-2-yn-1-yl benzoate (5f):

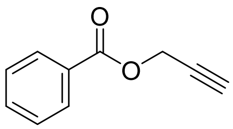

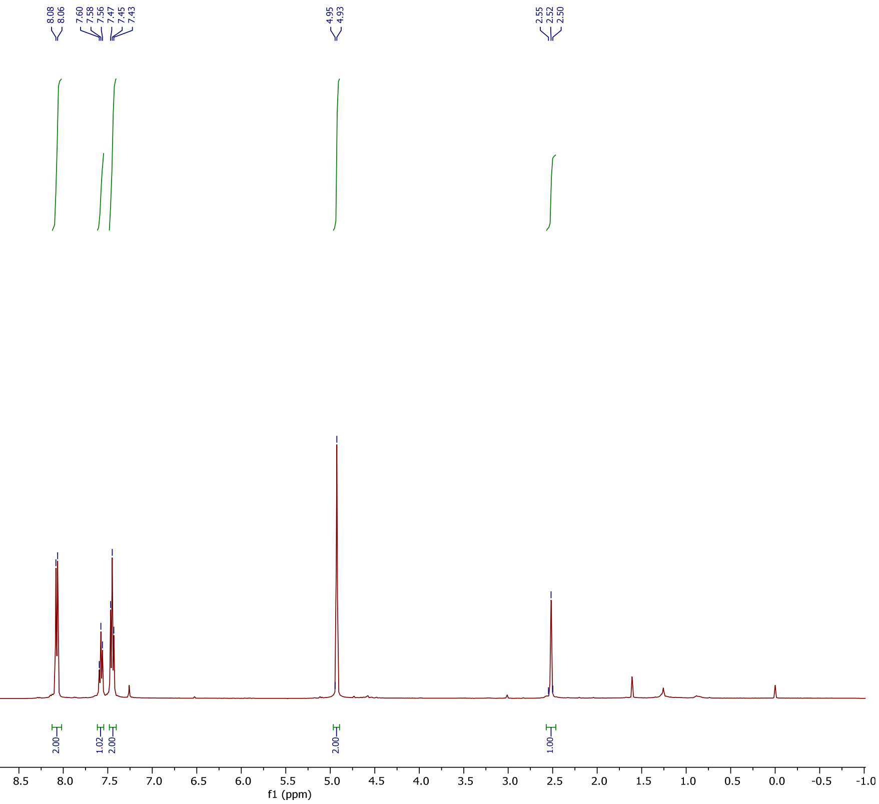

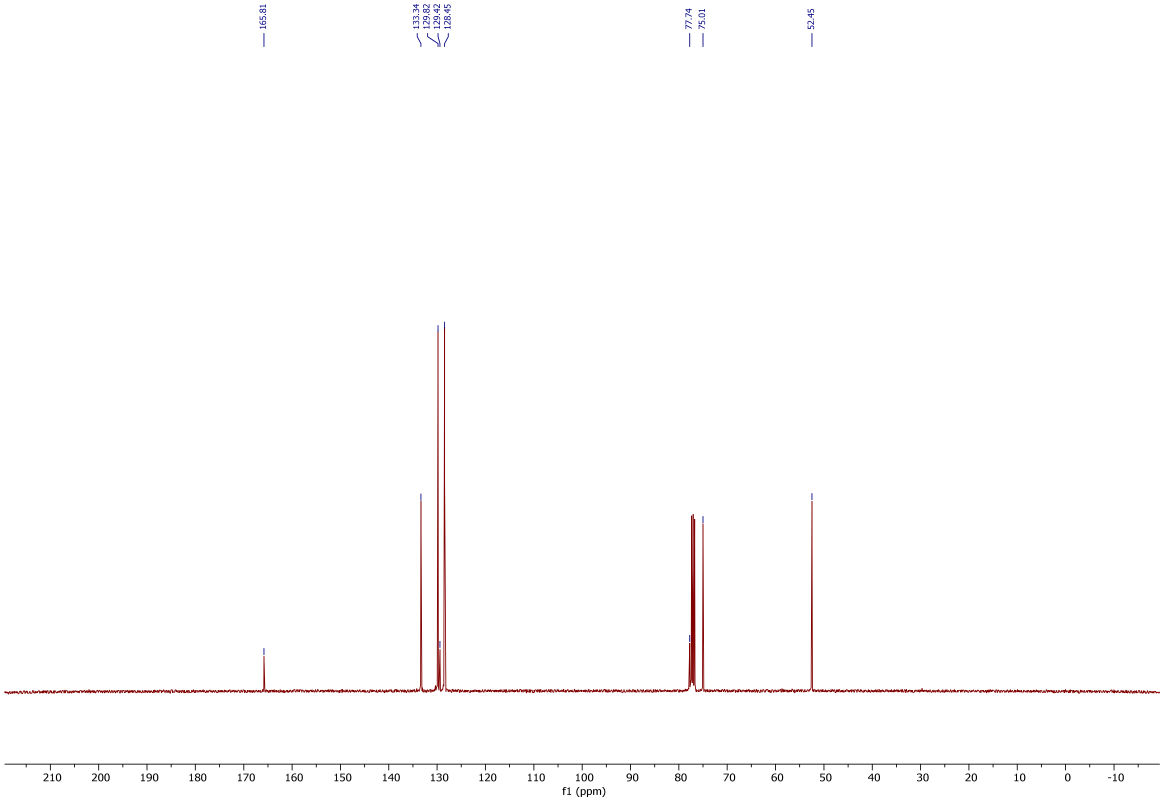

***Sec*-butyl benzoate (5g):**

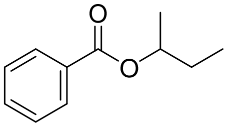

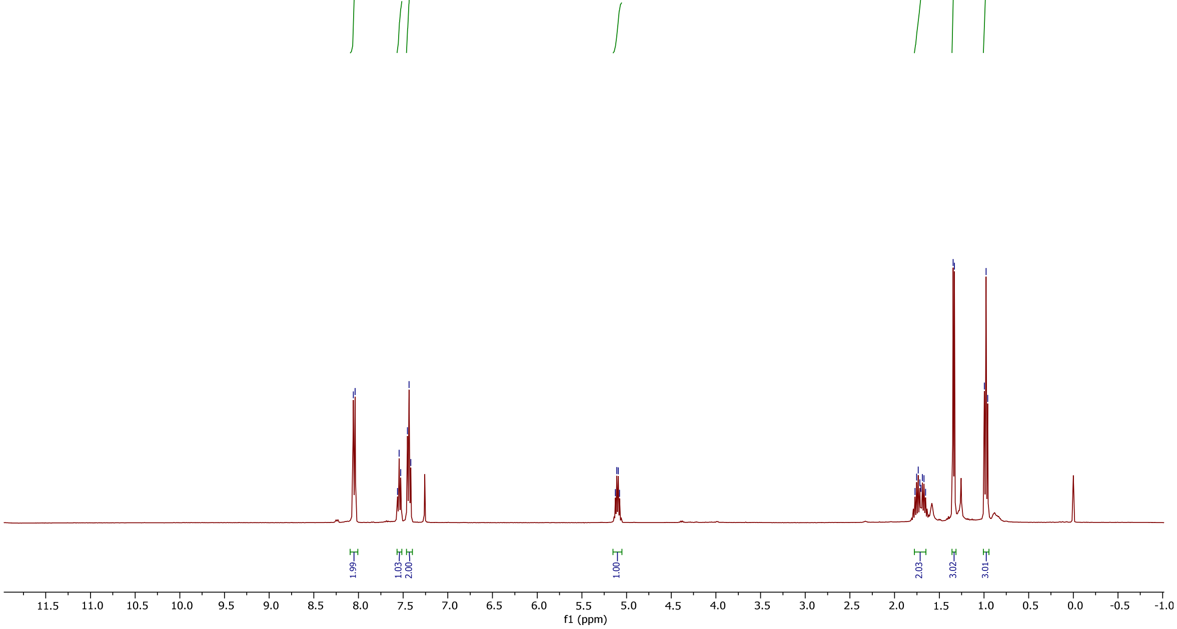

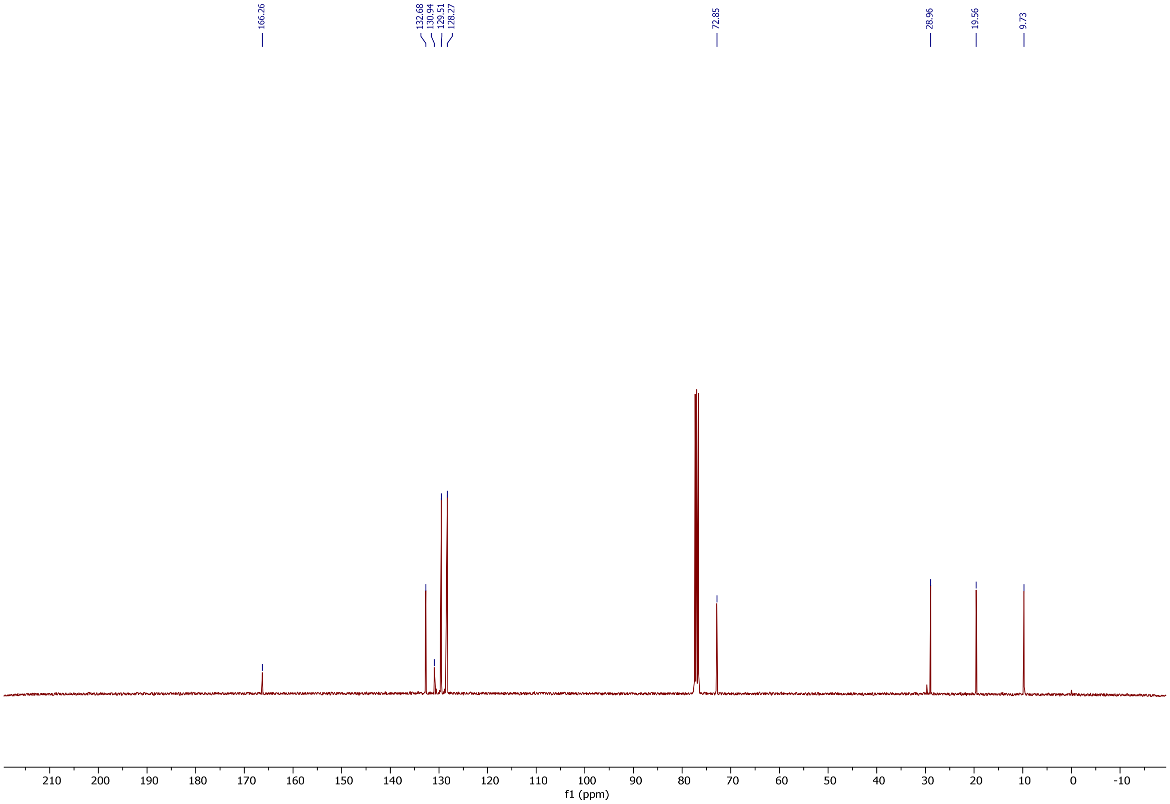

Benzyl 4-(*tert*-butyl)benzoate (6a):

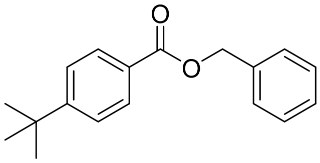

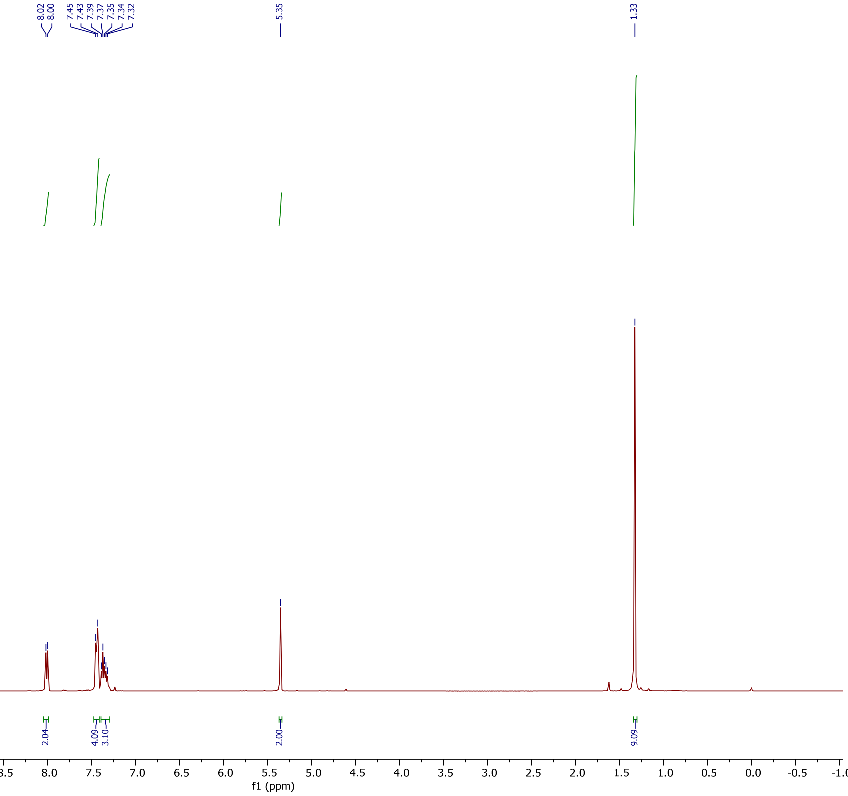

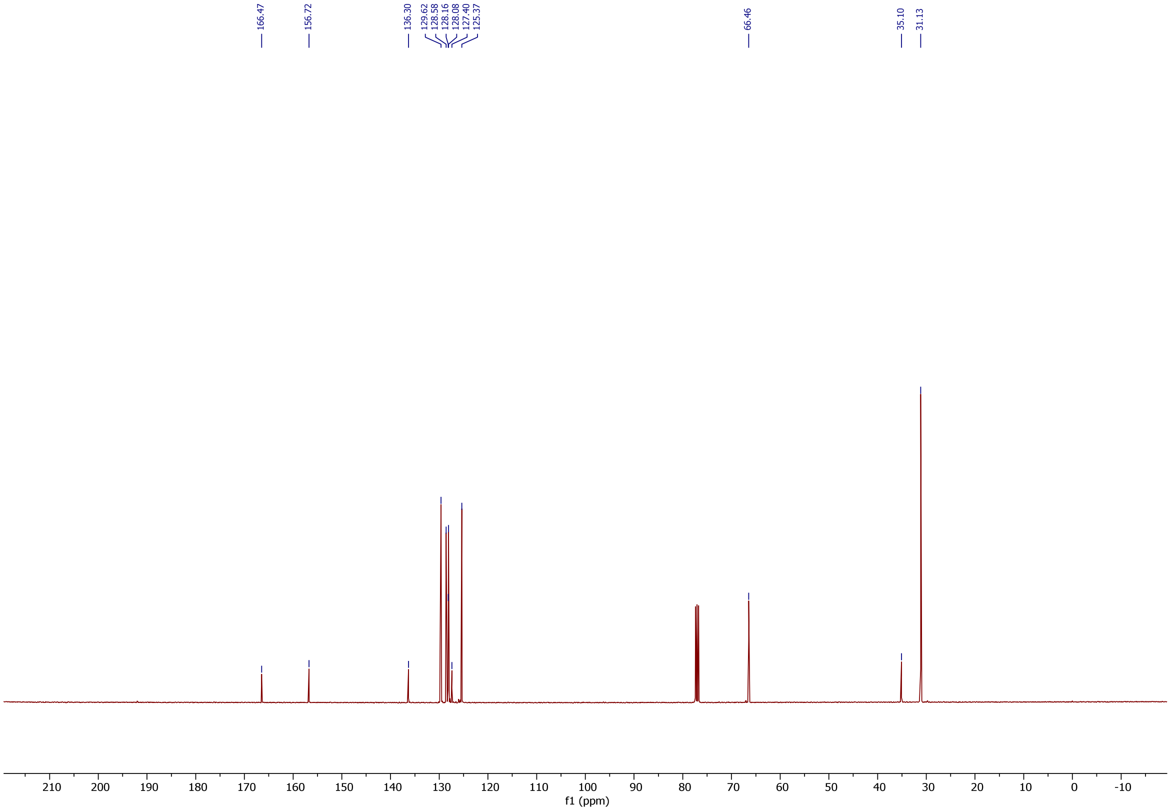

Benzyl [1,1'-biphenyl]-4-carboxylate (6b):

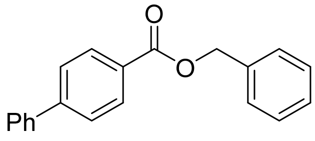

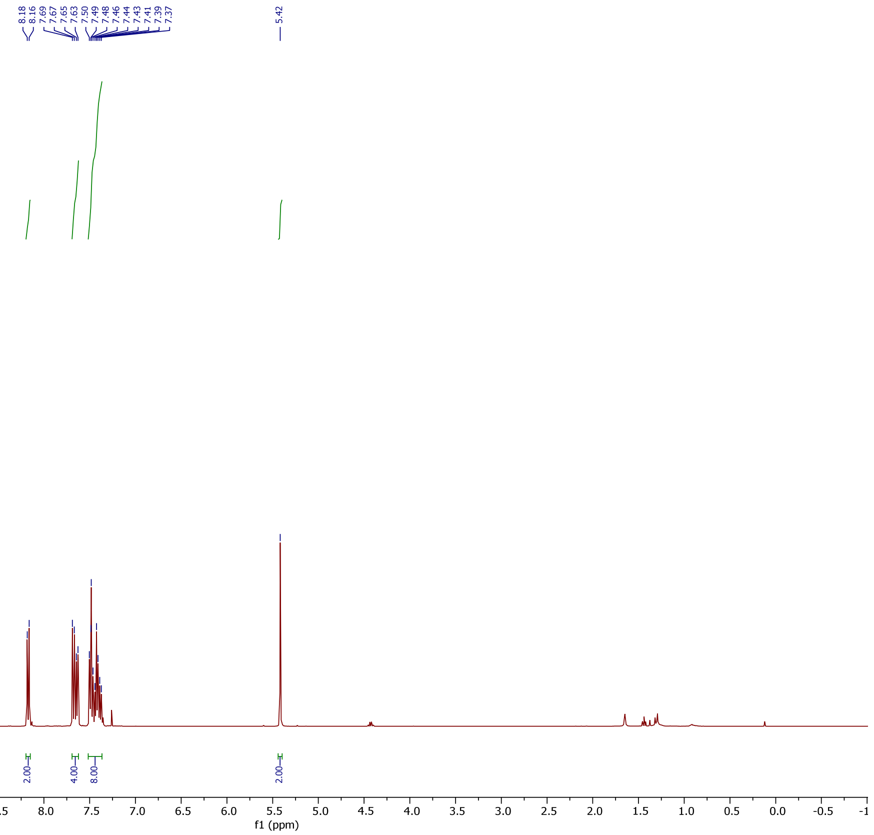

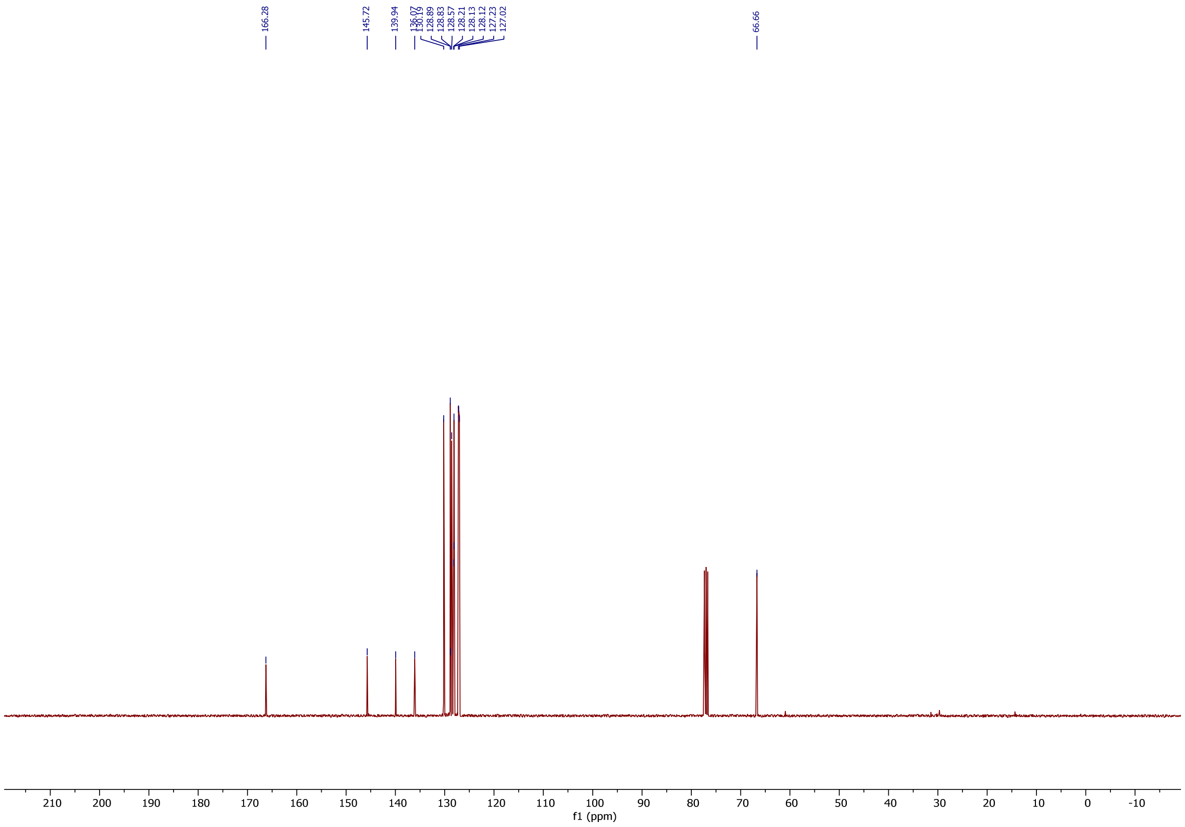

Benzyl 4-chlorobenzoate (6c):

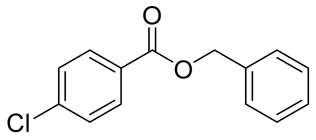

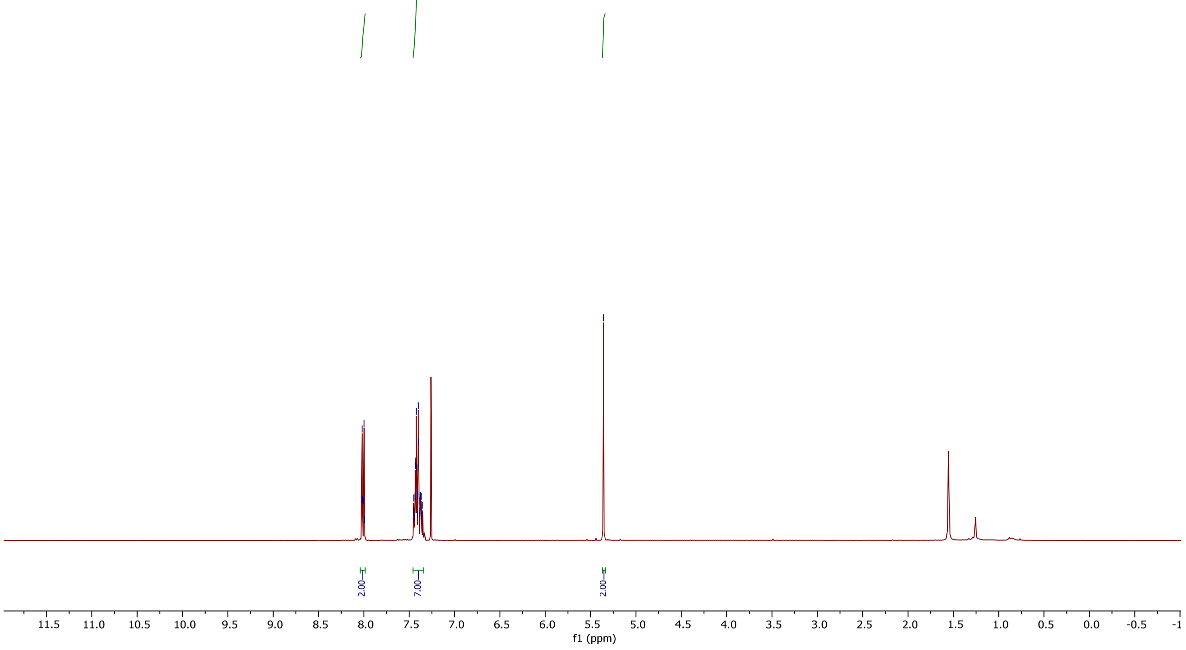

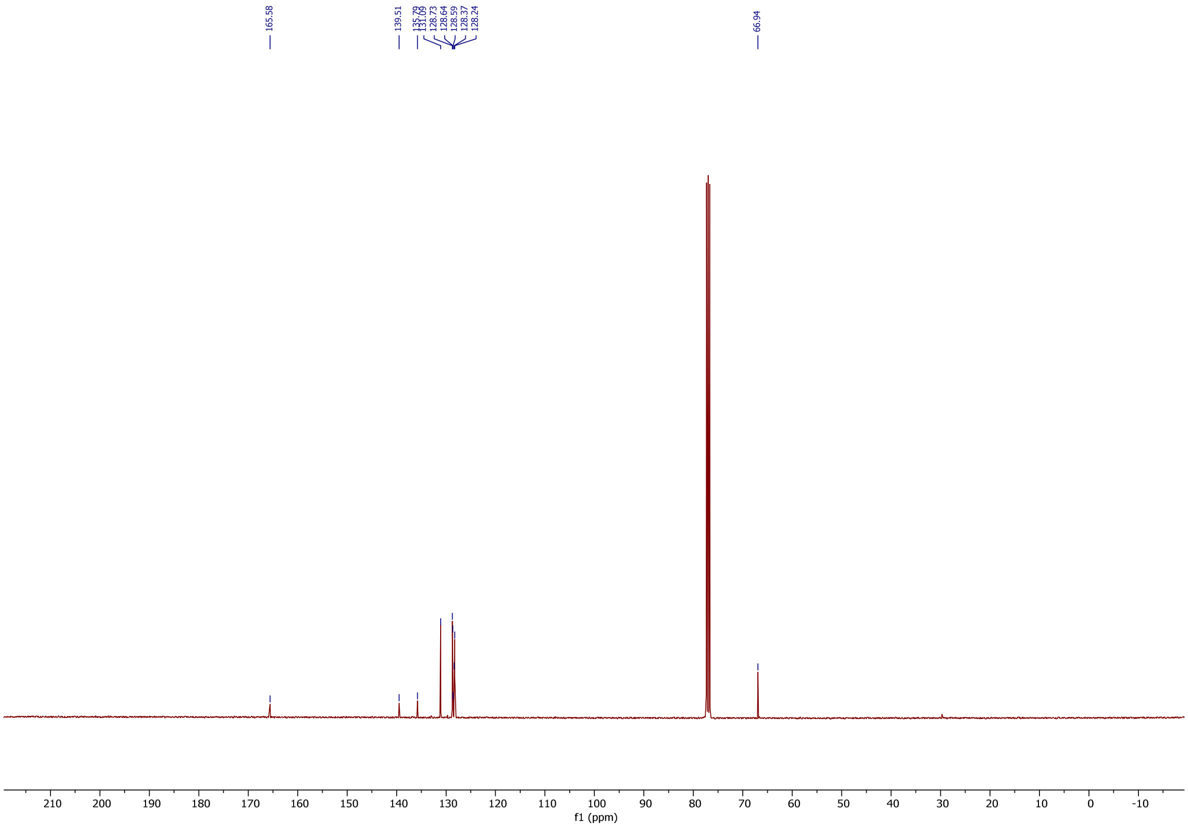

Benzyl 3-chlorobenzoate (6d):

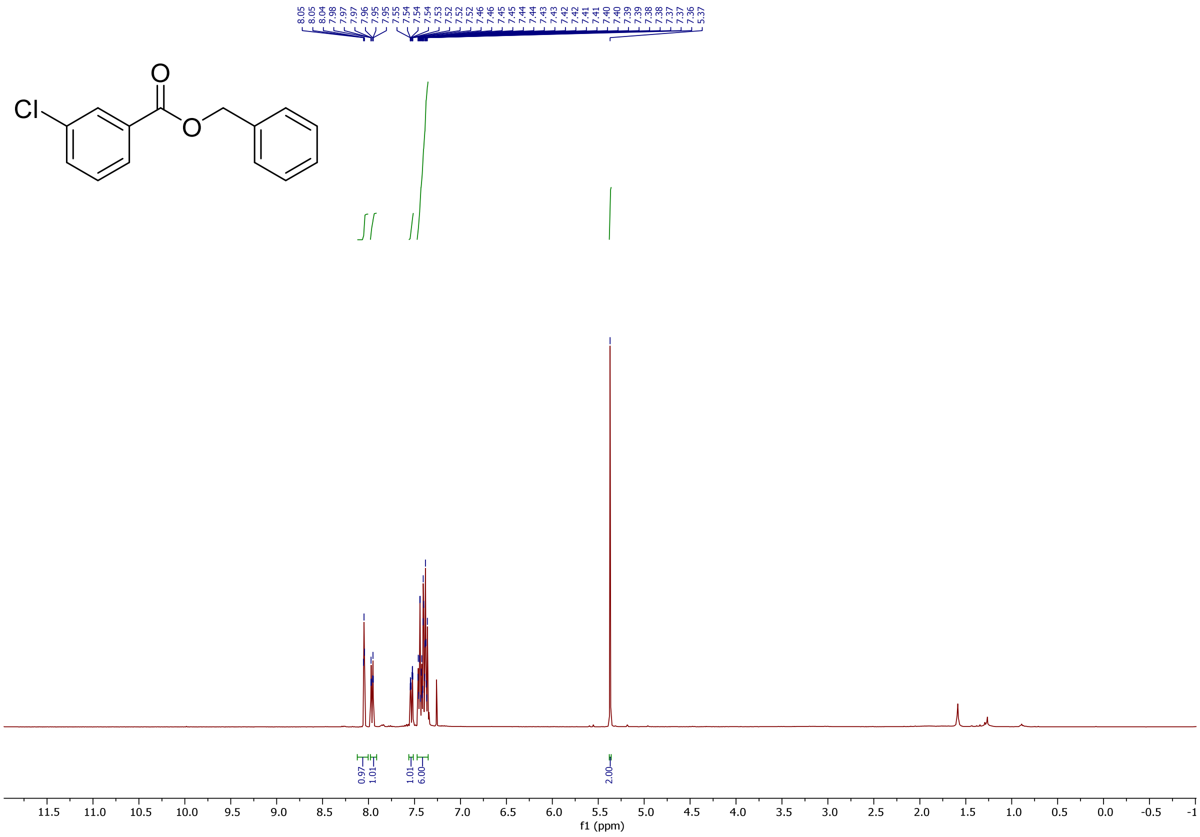

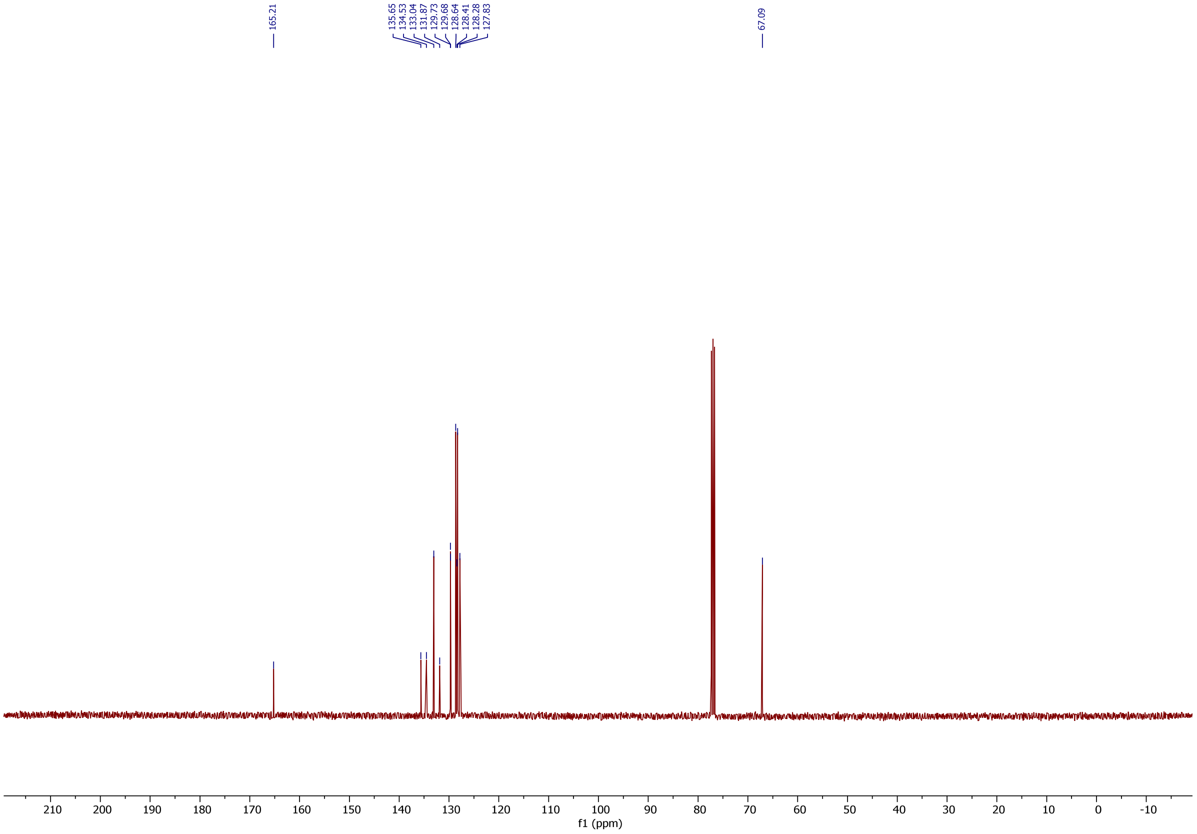

Benzyl 2,4-dichlorobenzoate (6e):

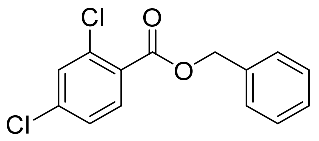

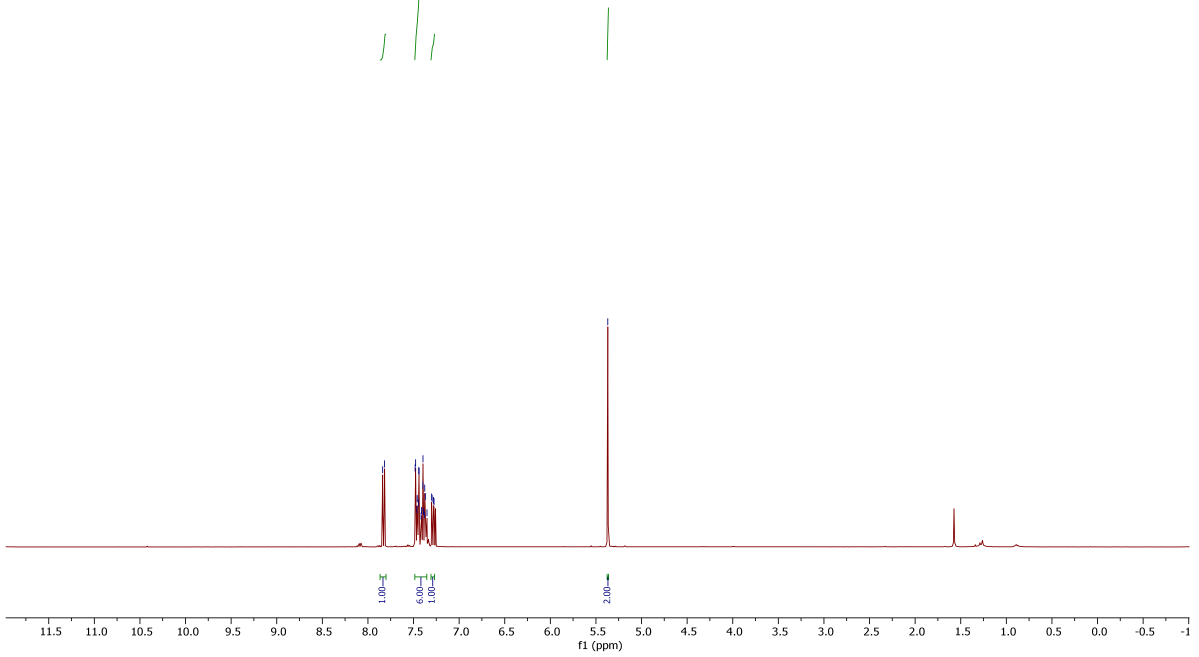

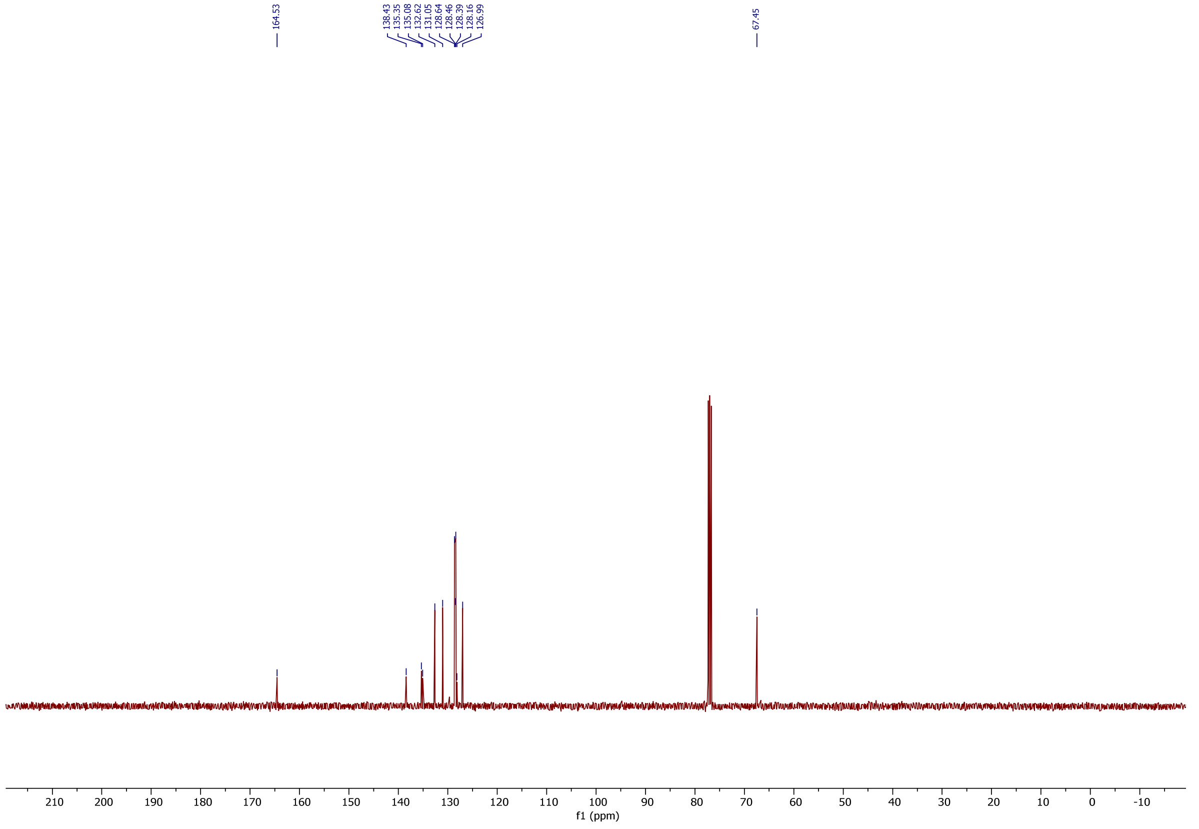

Benzyl 4-(trifluoromethyl)benzoate (6f):

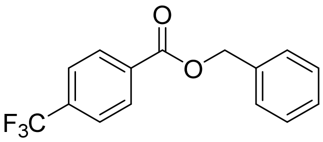

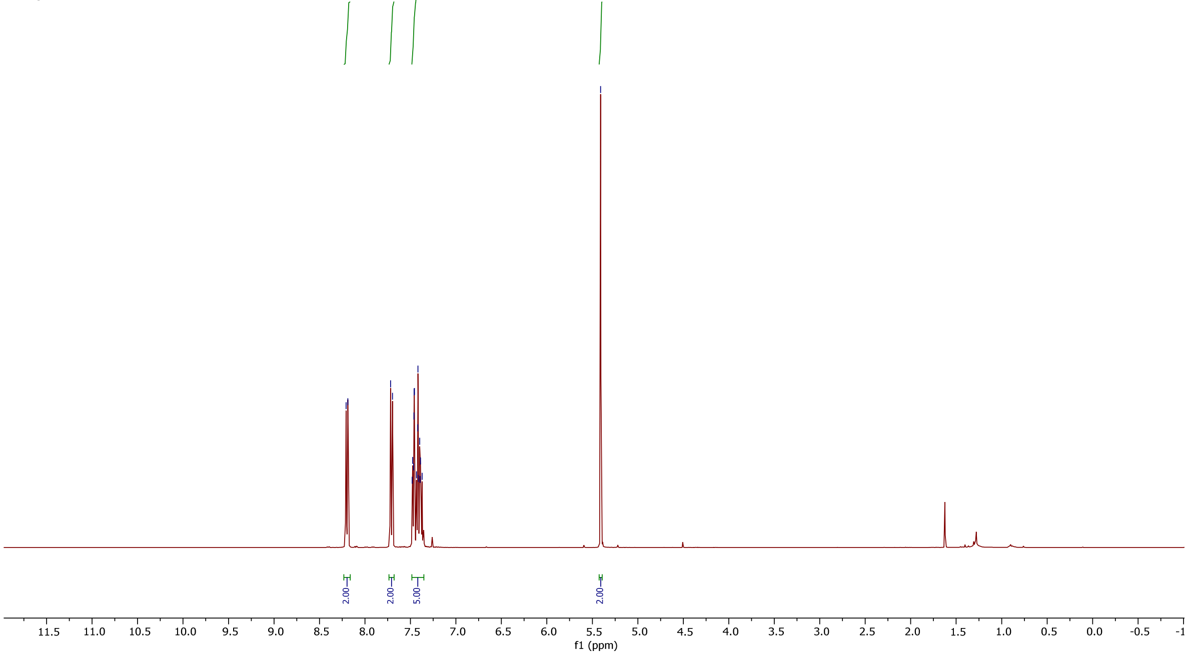

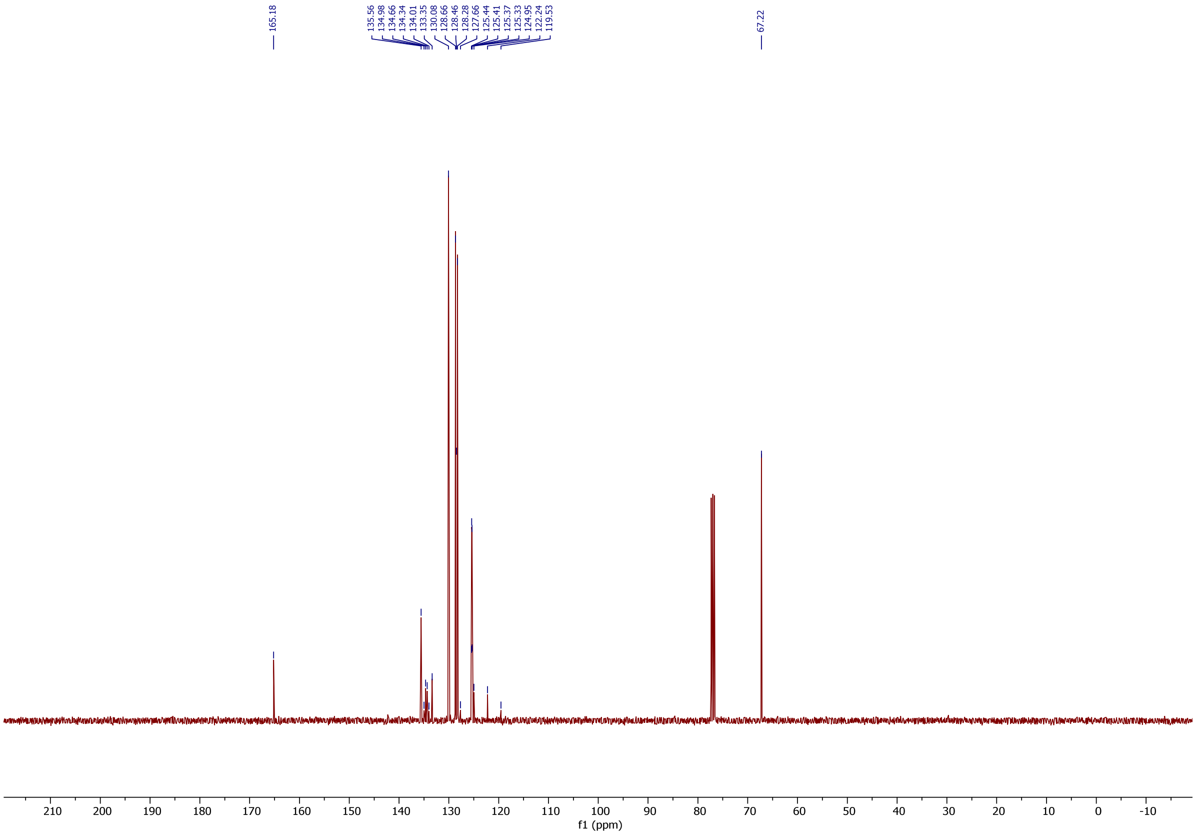

Benzyl 4-nitrobenzoate (6g):

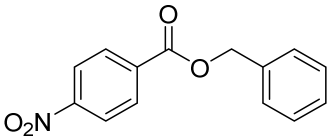

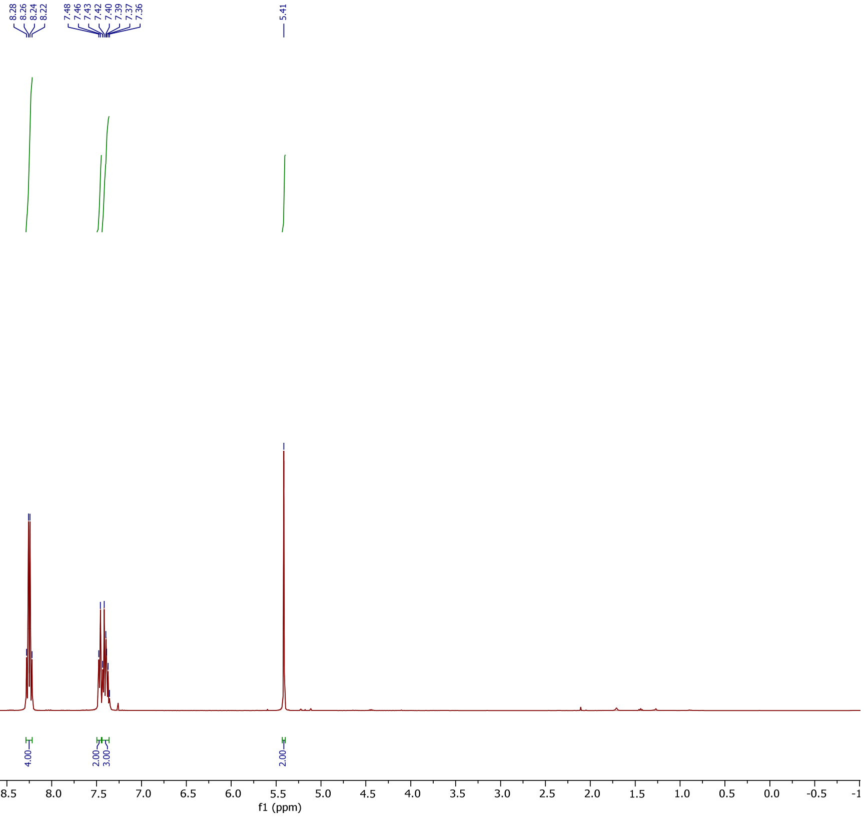

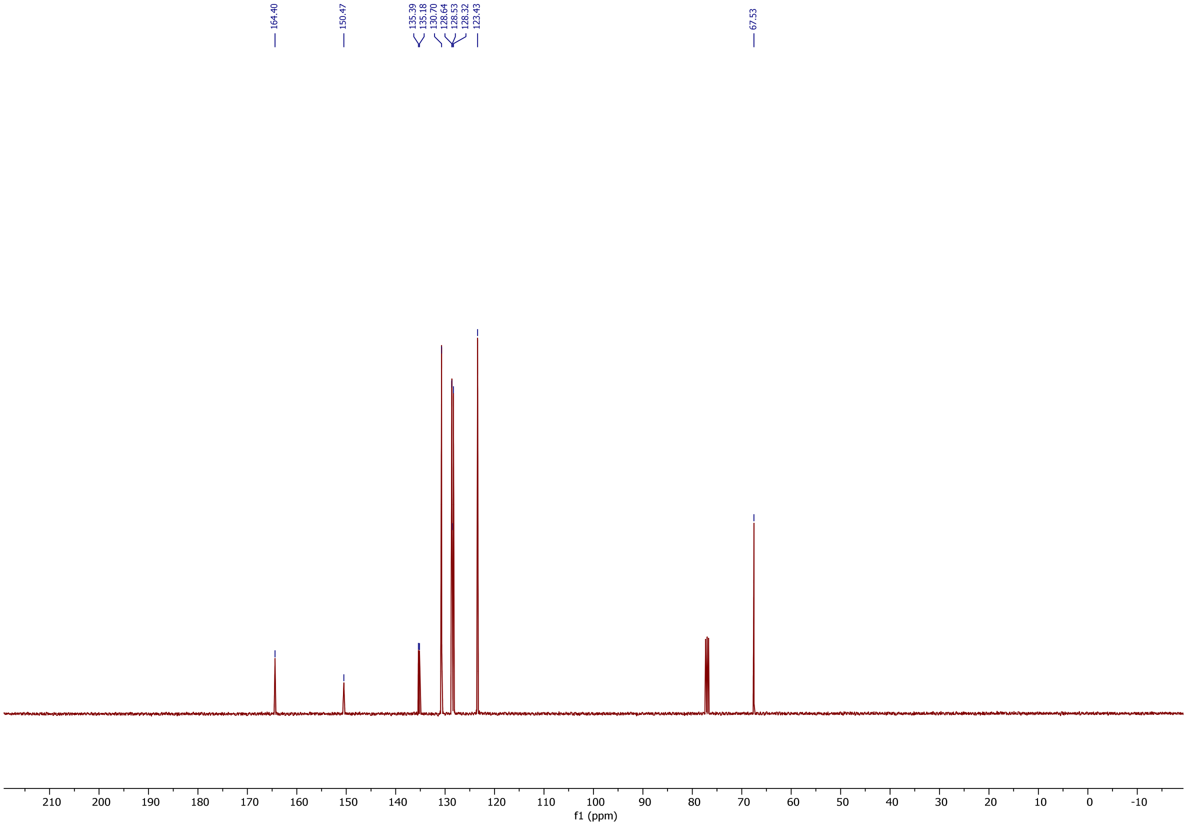

Methyl [1,1'-biphenyl]-4-carboxylate (7a):

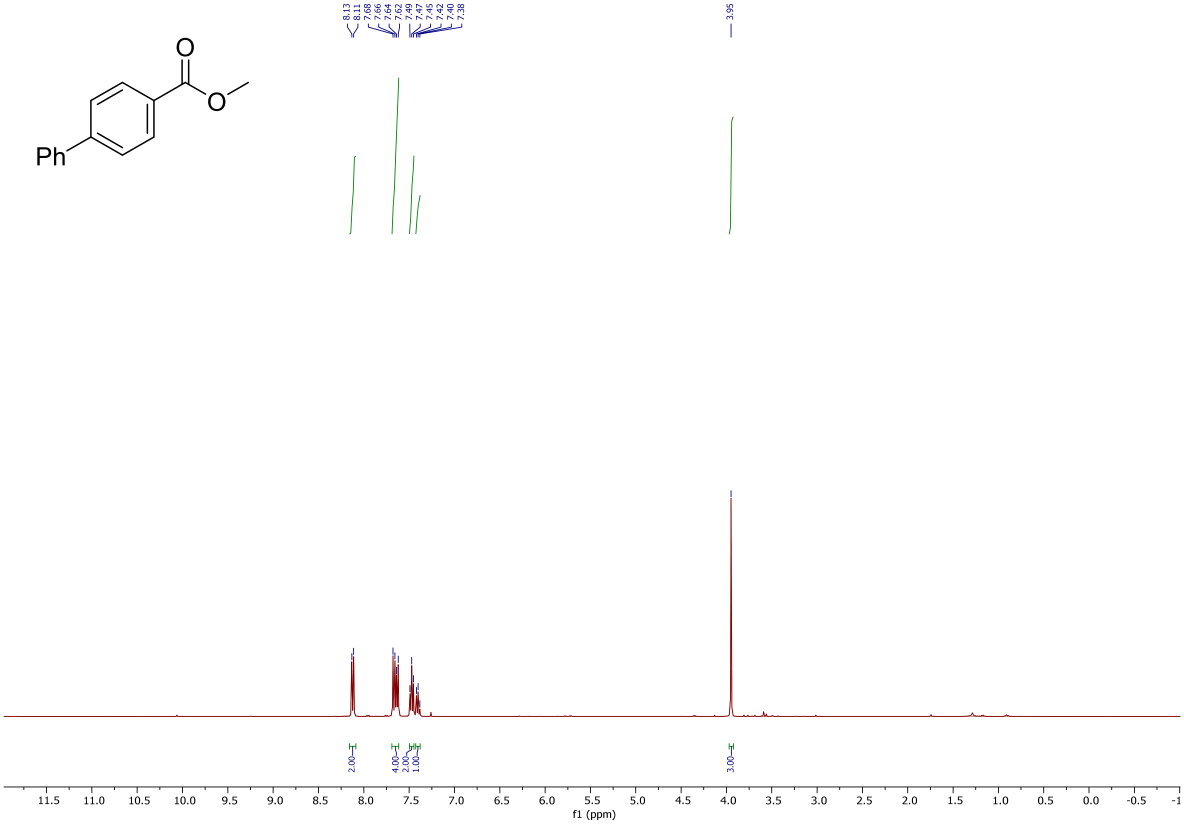

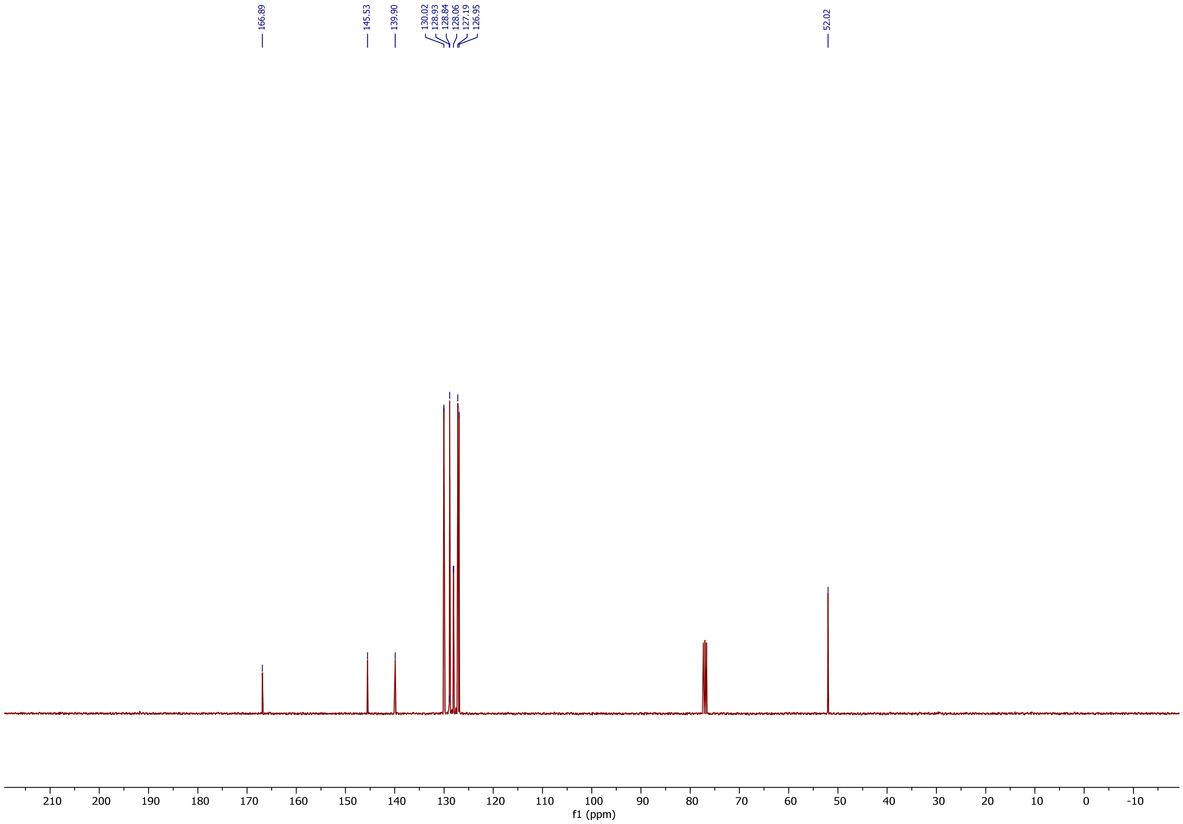

3-cyclohexylpropyl [1,1'-biphenyl]-4-carboxylate (7b):

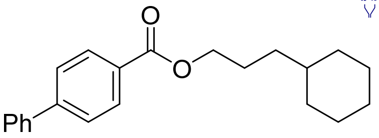

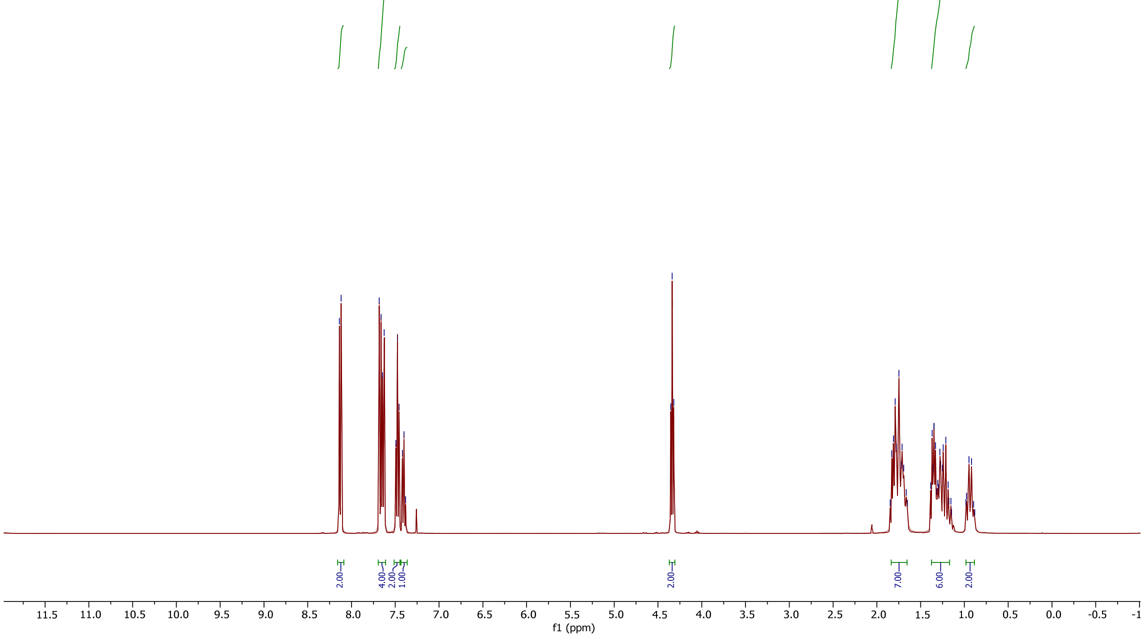

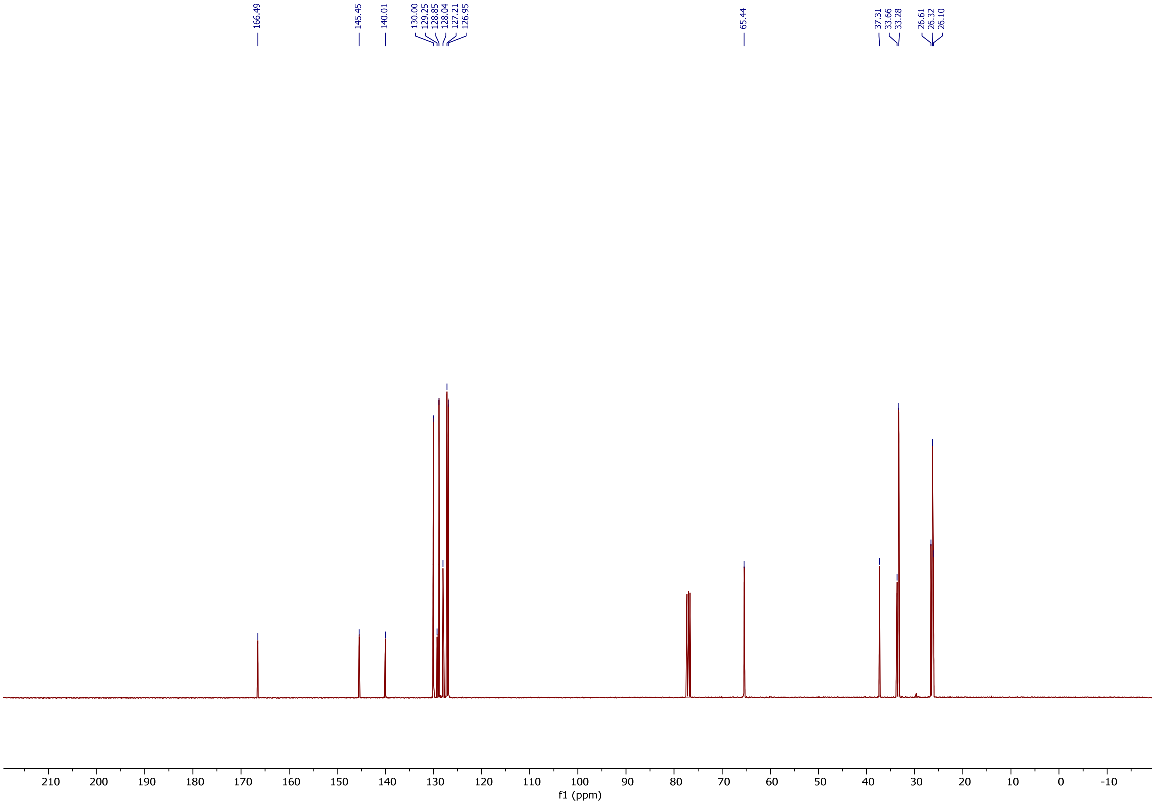

5-phenylpentyl 4-fluorobenzoate (7c):

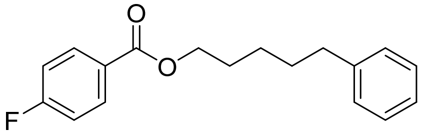

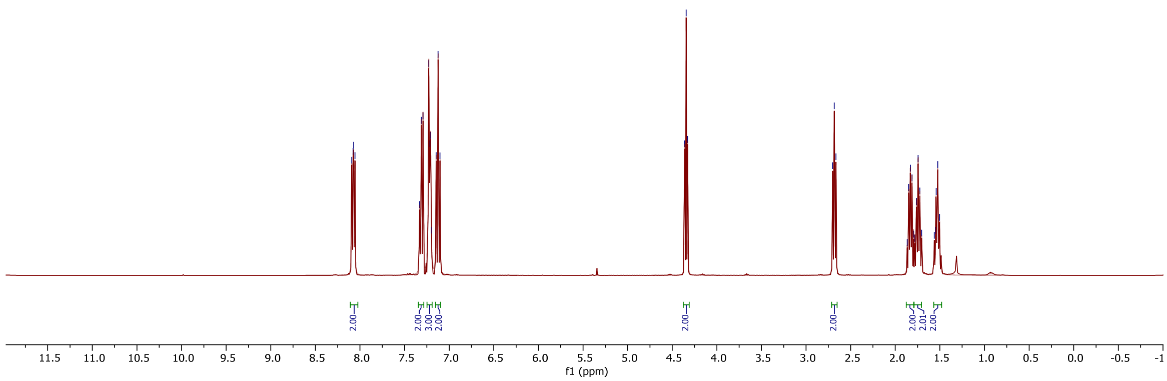

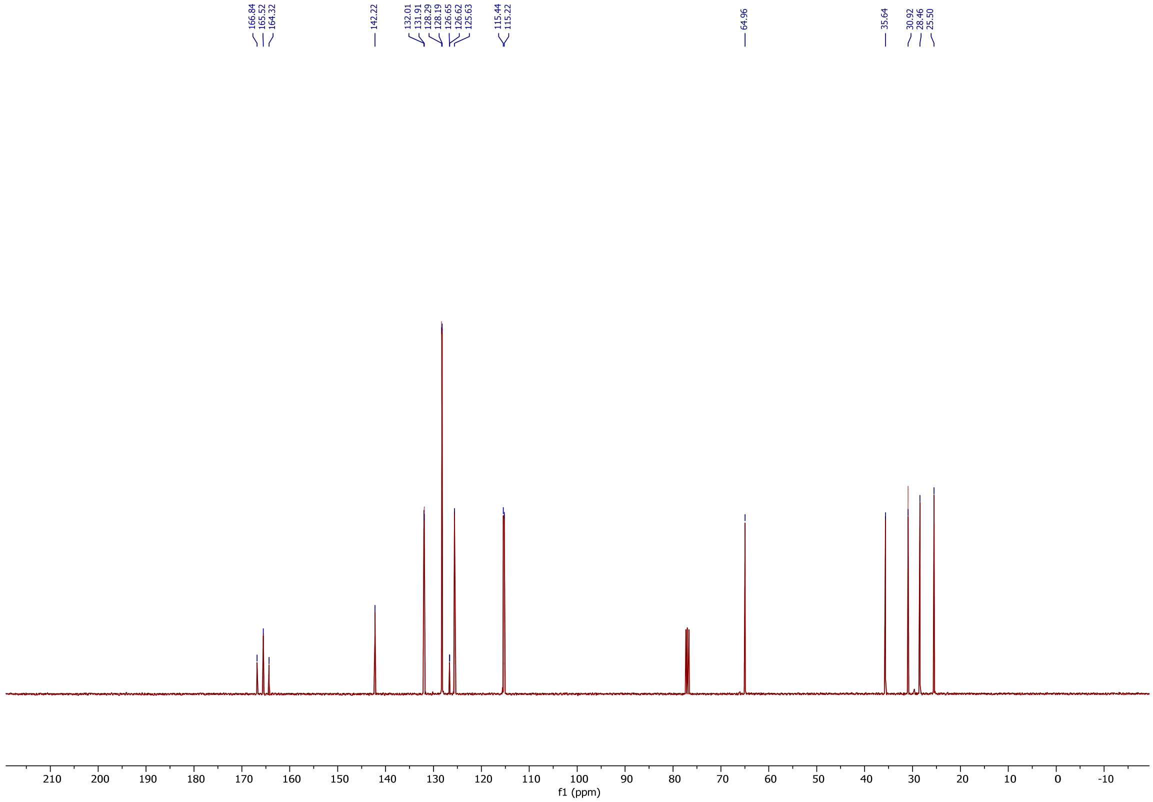

Ethyl 4-chlorobenzoate (7d):

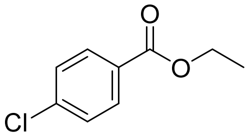

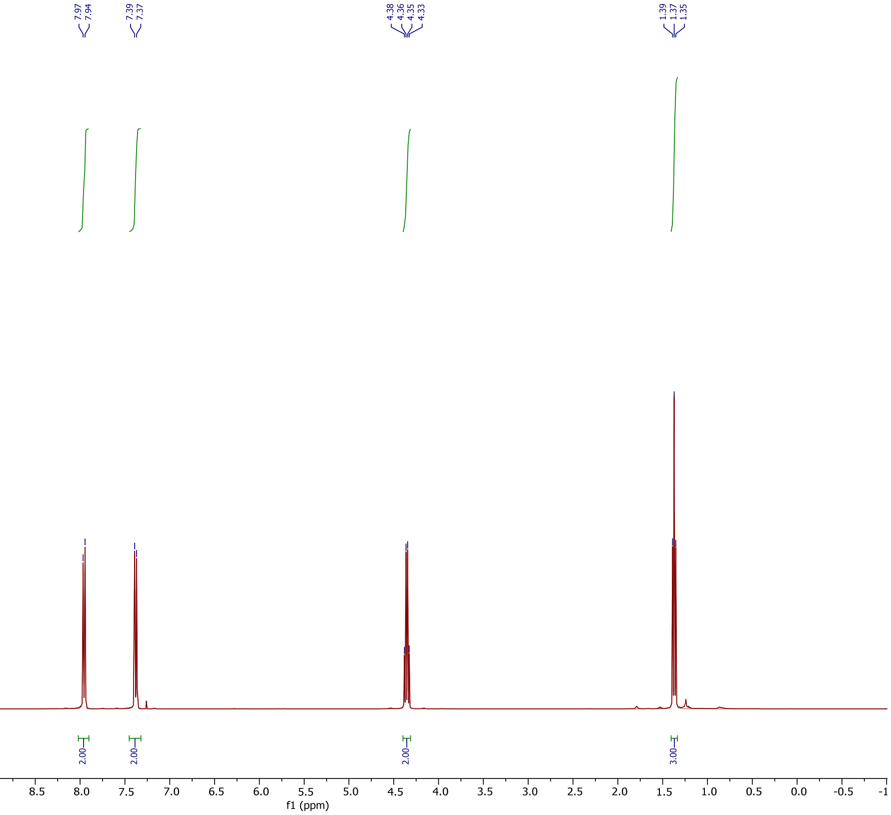

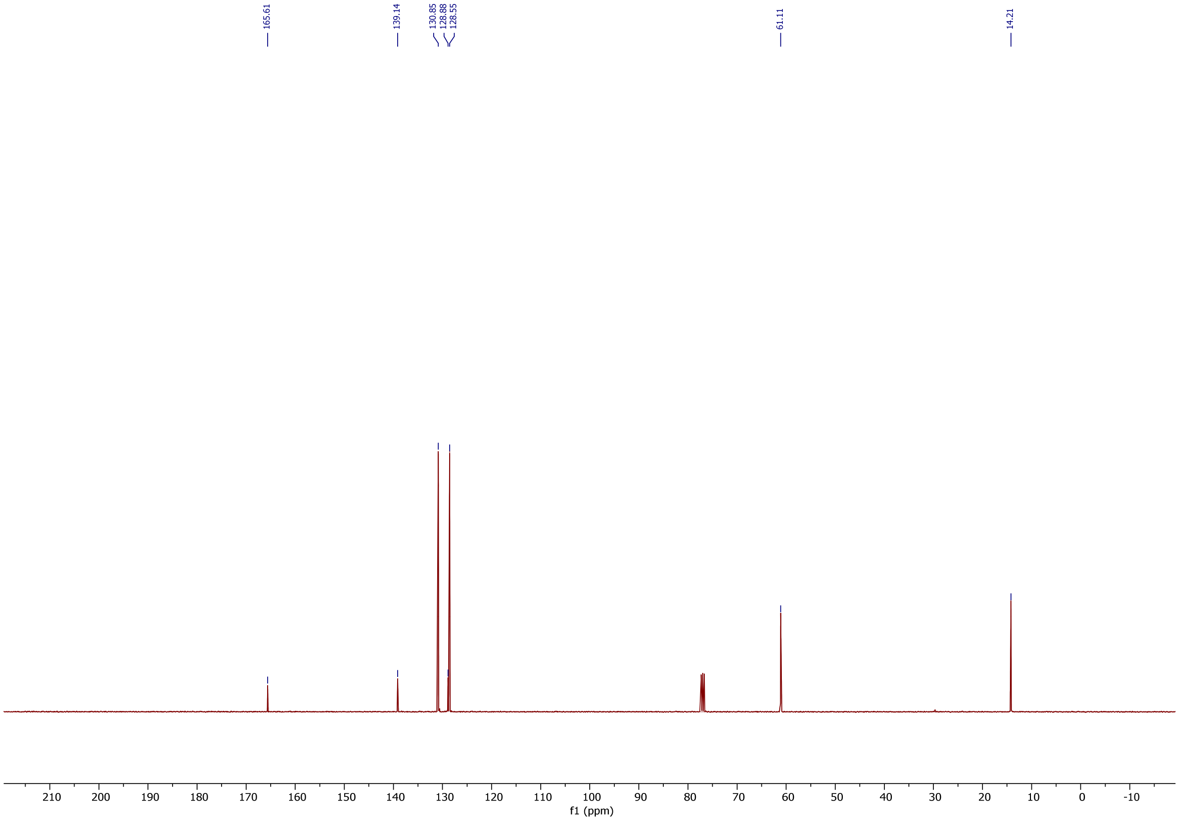

4-ethylphenyl 4-chlorobenzoate (7e):

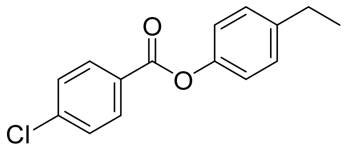

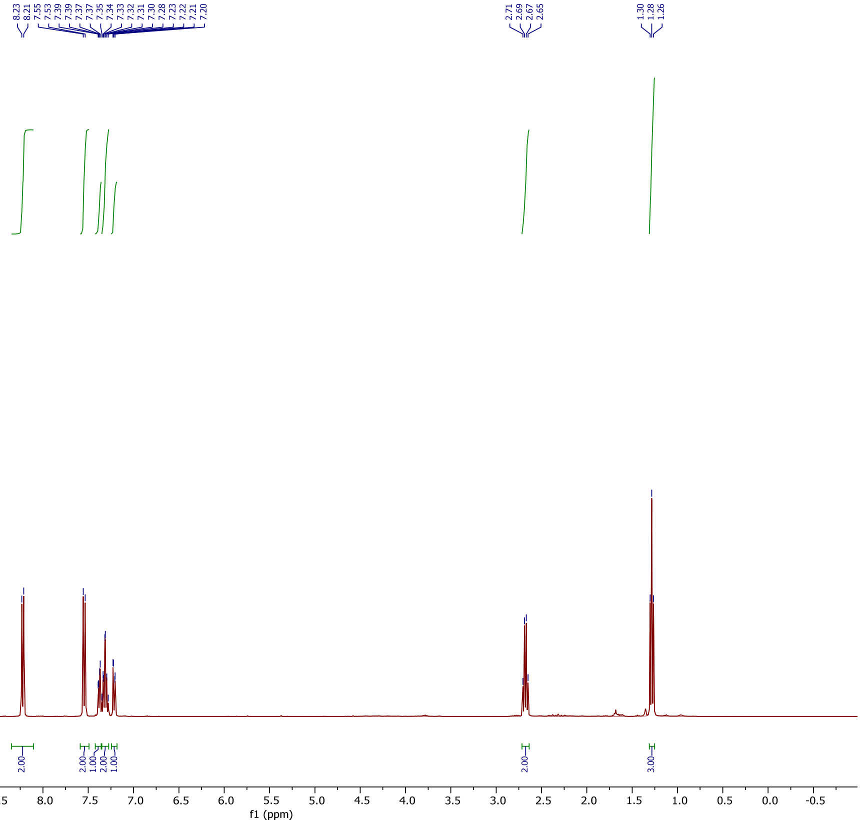

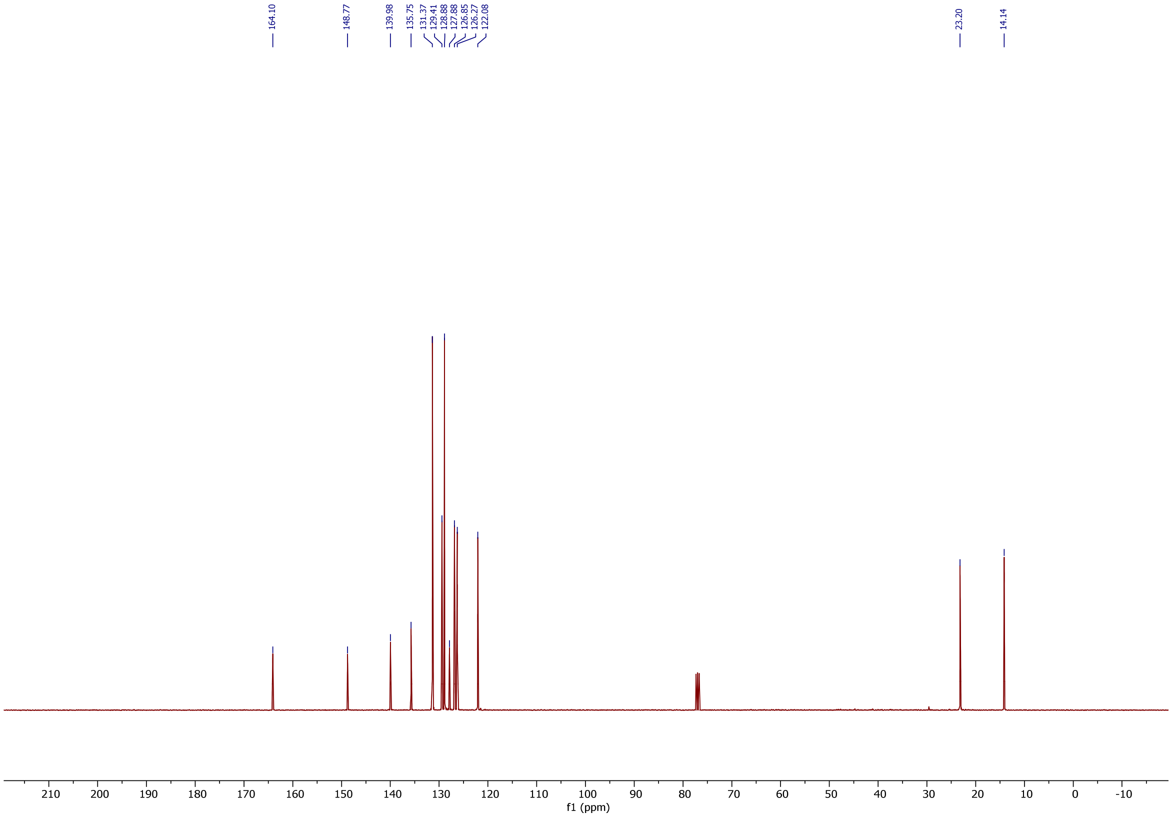

Thiophen-2-ylmethyl 3-chlorobenzoate (7f):

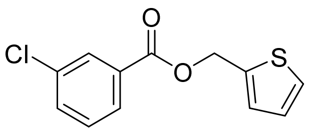

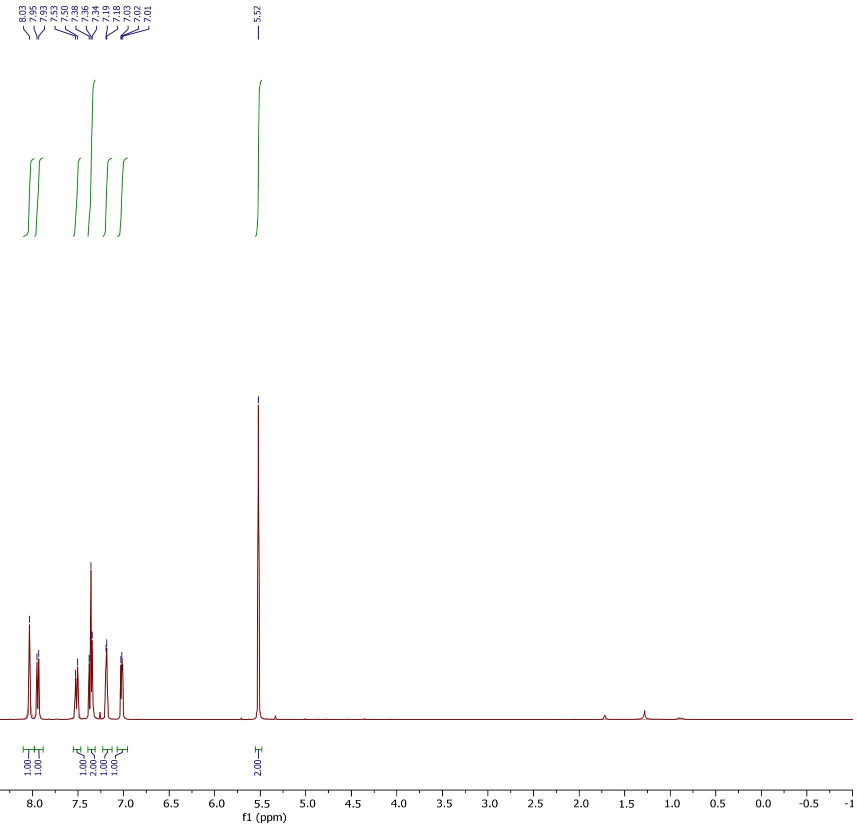

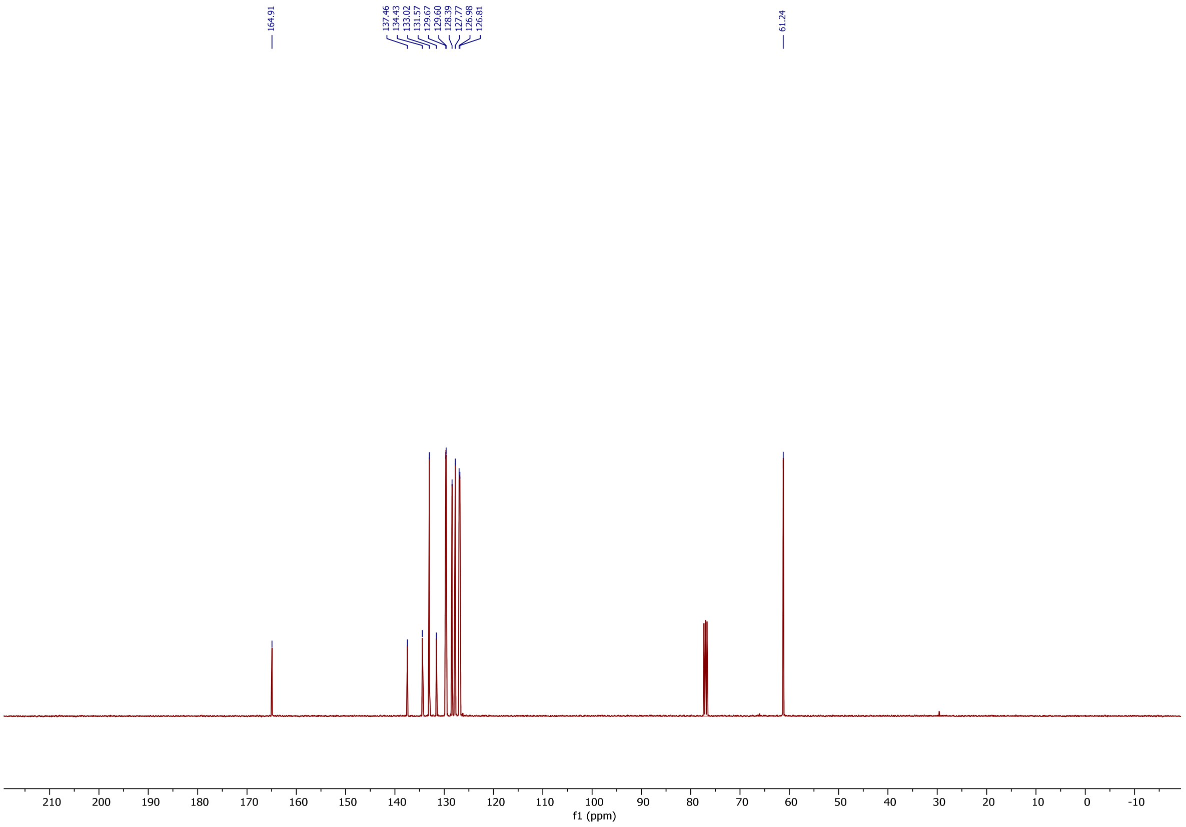

2.1. NMR Spectra of Benzoyl chloride (3):

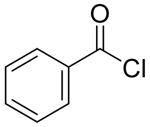

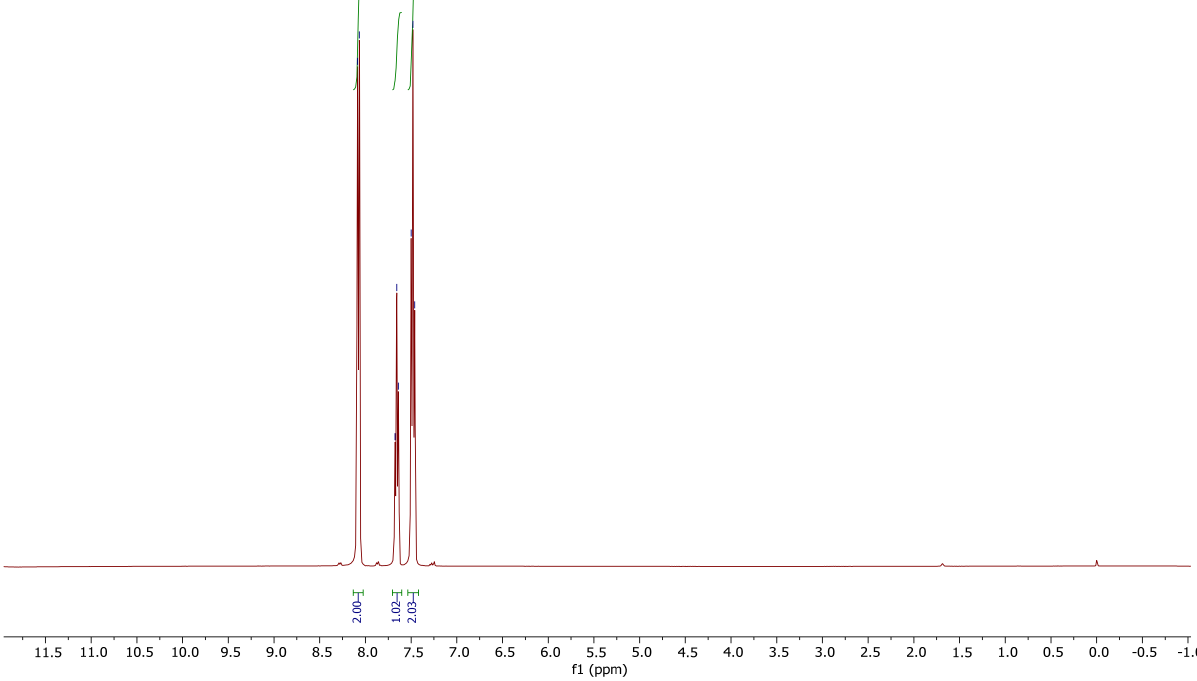

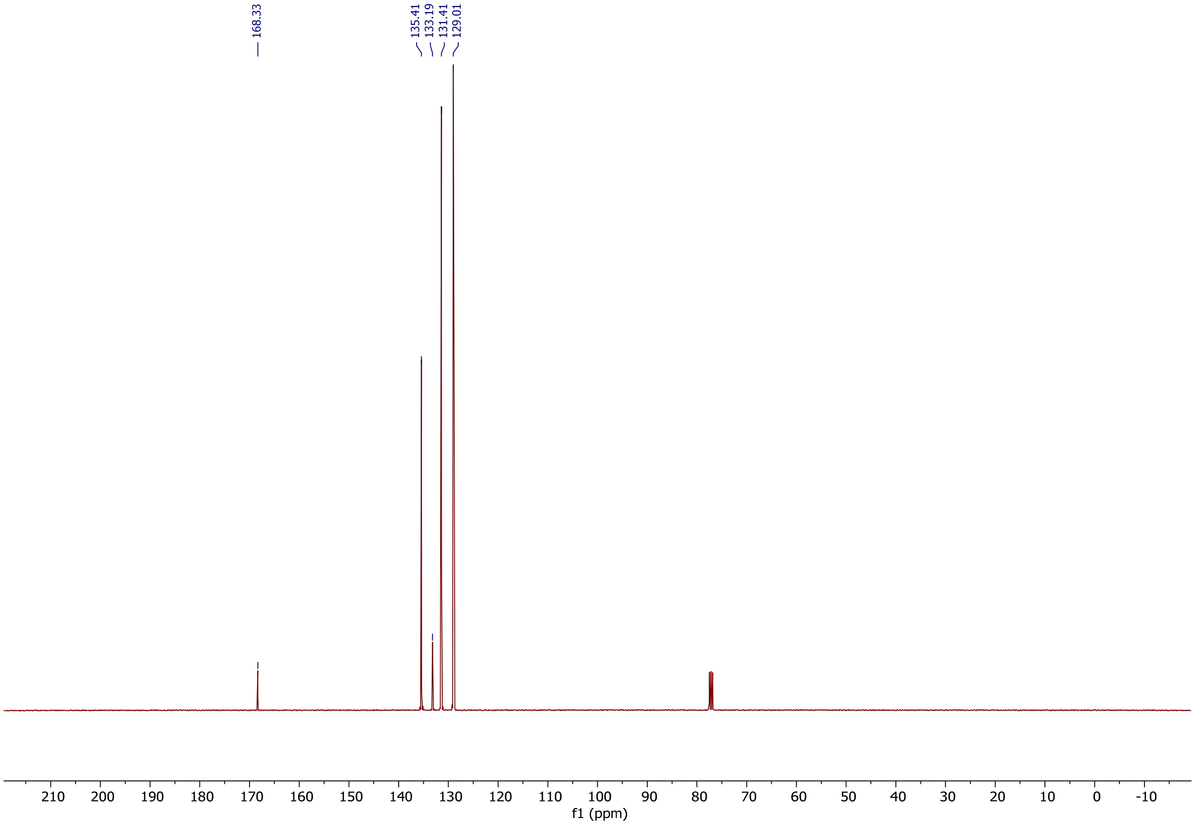

3. UV-Vis Spectra

TCCA (1.1 mmol) in 10 mL dichloromethane and benzyl alcohol (1.1 mmol).

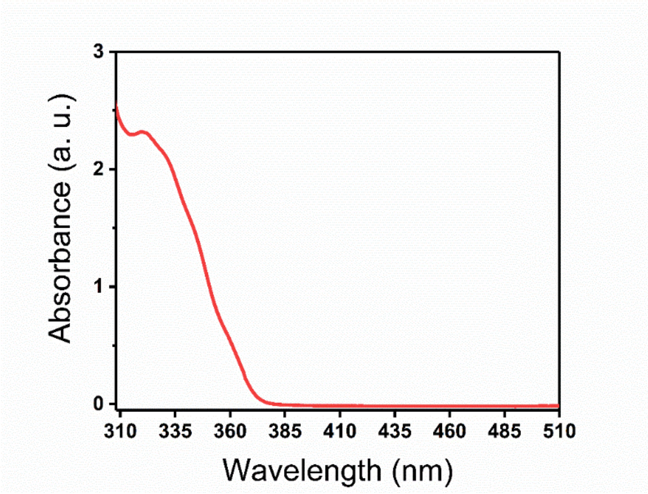

4. Experimental set-up:

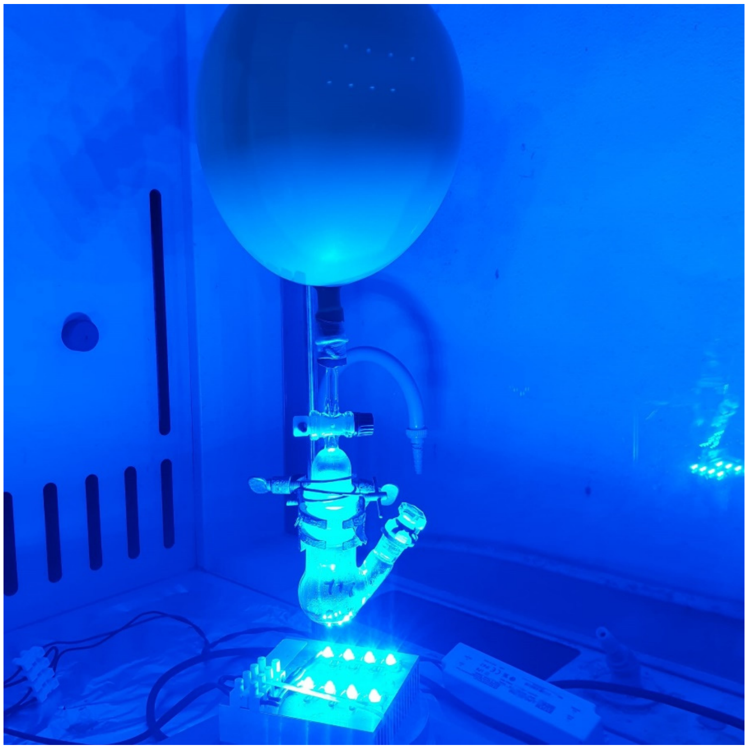

Supplement: Supplementary file 1 [file molecules-29-00570-s001.zip › molecules-2765757-supplementary.pdf]
